# Caldendrin–Jacob: A Protein Liaison That Couples NMDA Receptor Signalling to the Nucleus

**DOI:** 10.1371/journal.pbio.0060034

**Published:** 2008-02-26

**Authors:** Daniela C Dieterich, Anna Karpova, Marina Mikhaylova, Irina Zdobnova, Imbritt König, Marco Landwehr, Martin Kreutz, Karl-Heinz Smalla, Karin Richter, Peter Landgraf, Carsten Reissner, Tobias M Boeckers, Werner Zuschratter, Christina Spilker, Constanze I Seidenbecher, Craig C Garner, Eckart D Gundelfinger, Michael R Kreutz

**Affiliations:** 1 AG Molecular Mechanisms of Plasticity, Department of Neurochemistry and Molecular Biology, Leibniz-Institute for Neurobiology, Magdeburg, Germany; 2 Institute for Medical Neurobiology, Otto-von-Guericke University, Magdeburg, Germany; 3 Institute for Anatomy and Cell Biology, University of Ulm, Ulm, Germany; 4 Department of Psychiatry and Behavioral Sciences, Nancy Pritzker Laboratory, Stanford University, Palo Alto, California, United States of America; Wellcome Trust Sanger Institute, United Kingdom

## Abstract

NMDA (N-methyl-D-aspartate) receptors and calcium can exert multiple and very divergent effects within neuronal cells, thereby impacting opposing occurrences such as synaptic plasticity and neuronal degeneration. The neuronal Ca^2+^ sensor Caldendrin is a postsynaptic density component with high similarity to calmodulin. Jacob, a recently identified Caldendrin binding partner, is a novel protein abundantly expressed in limbic brain and cerebral cortex. Strictly depending upon activation of NMDA-type glutamate receptors, Jacob is recruited to neuronal nuclei, resulting in a rapid stripping of synaptic contacts and in a drastically altered morphology of the dendritic tree. Jacob's nuclear trafficking from distal dendrites crucially requires the classical Importin pathway. Caldendrin binds to Jacob's nuclear localization signal in a Ca^2+^-dependent manner, thereby controlling Jacob's extranuclear localization by competing with the binding of Importin-α to Jacob's nuclear localization signal. This competition requires sustained synapto-dendritic Ca^2+^ levels, which presumably cannot be achieved by activation of extrasynaptic NMDA receptors, but are confined to Ca^2+^ microdomains such as postsynaptic spines. Extrasynaptic NMDA receptors, as opposed to their synaptic counterparts, trigger the cAMP response element-binding protein (CREB) shut-off pathway, and cell death. We found that nuclear knockdown of Jacob prevents CREB shut-off after extrasynaptic NMDA receptor activation, whereas its nuclear overexpression induces CREB shut-off without NMDA receptor stimulation. Importantly, nuclear knockdown of Jacob attenuates NMDA-induced loss of synaptic contacts, and neuronal degeneration. This defines a novel mechanism of synapse-to-nucleus communication via a synaptic Ca^2+^-sensor protein, which links the activity of NMDA receptors to nuclear signalling events involved in modelling synapto-dendritic input and NMDA receptor–induced cellular degeneration.

## Author Summary

Long-lasting changes in communication between nerve cells require the regulation of gene expression. The influx of calcium ions into the cell, particularly through membrane protein called NMDA receptors, plays a crucial role in this process by determining the type of gene expression induced. NMDA receptors can exert multiple and very divergent effects within neuronal cells by impacting opposing phenomena such as synaptic plasticity and neuronal degeneration. We identified a protein termed Jacob that appears to play a pivotal role in such processes by entering the nucleus in response to NMDA receptor activation and controlling gene expression that governs cell survival and the stability of synaptic cell contacts. Removal of Jacob from the nucleus protects neurons from NMDA receptor–induced cell death and increases phosphorylation of the transcription factor CREB, whereas the opposite occurs after targeting Jacob exclusively to the nucleus. The work defines a novel pathway of synapse-to-nucleus communication involved in modelling synapto-dendritic input and NMDA receptor–induced cellular degeneration.

## Abstract 3

A new signaling mechanism from NMDA receptors to the nucleus plays an important role in the phosphorylation of the transcription factor CREB and neuronal cell survival.

## Introduction

Ca^2+^ signals triggered by NMDA-type glutamate receptors can result in long-lasting changes of synaptic input and dendritic cytoarchitecture in phenomena commonly referred to as neuronal plasticity. On the contrary, NMDA receptors are also important players in neurodegenerative processes. Although both aspects require gene expression, our knowledge is still sparse concerning how these fundamental processes are regulated at the molecular level. The Janus face of neuronal NMDA receptor signalling is probably best reflected by the fact that the influx of Ca^2+^ ions is thought to act as one of the major mediators of synapto-nuclear signalling [1,2] and of excitotoxic cell death [3]. Within this scheme, a prevailing idea is the existence of Ca^2+^ microdomains coupled to the activation of synaptic and extrasynaptic NMDA receptors, and transducing incoming Ca^2+^ events to different downstream pathways [1–4]. In a series of elegant studies, Hardingham and colleagues [5–7] provided evidence that Ca^2+^ influx through synaptic NMDA receptors trigger nuclear cAMP response element-binding protein (CREB) phosphorylation via an extracellular signal-regulated kinase (ERK)-dependent pathway, whereas Ca^2+^ influx through extrasynaptic NMDA receptors leads via an ERK-independent pathway to a dephosphorylation of CREB termed CREB shut-off. As opposed to the synaptic pathway, the CREB shut-off signal is coupled to neuronal degeneration and cell death [7]. Thus, CREB-regulated gene expression appears to be a shared mechanism for both long-term plasticity and neuronal survival [1–3,8–10].

Although Ca^2+^ exerts its signalling functions via a variety of Ca^2+^ sensor proteins, pathways that result in a nuclear response to synaptic activity have primarily been based on signalling via calmodulin (CaM) [1,2]. In its Ca^2+^-bound state, CaM alters the properties of several other proteins and signalling cascades that have been implicated in diverse neuronal functions [11,12]. Whereas it is tacitly assumed that CaM is present in large excess in all cellular compartments, and therefore regulation of CaM signalling largely depends on binding of Ca^2+^ ions, a variety of additional EF-hand proteins have been identified in neurons, termed neuronal Ca^2+^ sensor (NCS) proteins. These NCS are believed to serve more-specific functions in neurons [13–15].

One of these NCS proteins is Caldendrin (also termed CaBP1) [16,17], a bipartite protein with a unique N-terminal half and a C-terminal half that contains four EF-hand motifs and qualifies Caldendrin as the closest relative of CaM in brain neurons. The second EF-hand is most likely cryptic [16,18]. Modelling of the C-terminal segment suggests that Caldendrin displays an altered surface-exposed amino acid residue distribution, especially at EF-hand 2 as compared to CaM [18]. Interestingly, the unique N-terminal half of Caldendrin exhibits no similarity to other known proteins [16]. Moreover, in contrast to the ubiquitously expressed CaM, Caldendrin is only present in a subset of synapses and seems to be exclusively and tightly associated with the somatodendritic cytoskeleton and the postsynaptic density (PSD) of mature principal neurons in brain regions with a laminar organization [16,19]. To test the hypothesis that Caldendrin might have specific functions in neurons that are distinct from those of CaM, we performed a yeast two-hybrid screen to identify specific interaction partners for the C-terminal half of Caldendrin.

This strategy disclosed a novel Caldendrin-binding partner, named Jacob, which exhibits a remarkably restricted expression in cortical and limbic brain regions of mammals. We report that Jacob displays a distribution similar to that of Caldendrin in the PSD, dendritic spines, and dendrites, but in contrast to Caldendrin, is also found in neuronal nuclei. Activation of NMDA receptors induces nuclear trafficking of Jacob that is under the control of Caldendrin and Importin-α. Our data imply that nuclear Jacob participates in the CREB shut-off pathway, which might play a physiological as well as pathophysiological role in the control of dendritic cytoarchitecture, synapse number, and neuronal survival under conditions of increased NMDA receptor activity.

## Results

### Identification and Cloning of Jacob, a Caldendrin Binding Protein in Brain

**Figure 1:**
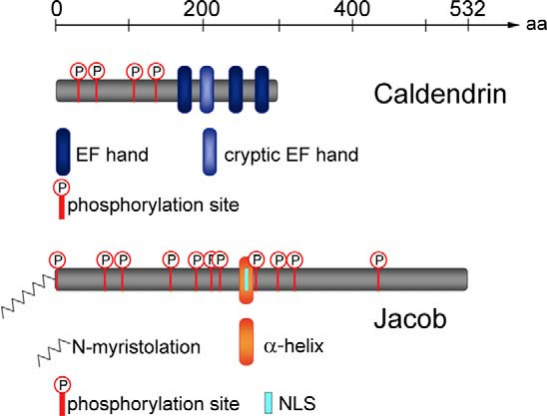
Primary Structures of Caldendrin and Jacob Schematic representation of Caldendrin (top) and Jacob (bottom) as predicted from their cDNA sequences. The main sequence features including potential phosphorylation sites, EF-hand structures, N-myristoylation, bipartite NLS, and central α-helical region are depicted.

Utilizing yeast two-hybrid screening to identify binding partners for Caldendrin, we obtained eight independent clones of a hitherto uncharacterized gene product, which we termed Jacob. The longest cDNA clone encompassed an open reading frame of 1,596 bp encoding a 532–amino acid (aa) protein with a calculated molecular weight of 60 kDa (Figures 1 and S1A). Several clones obtained from rat brain cDNA libraries reveal alternatively spliced Jacob transcripts (Figure S1B), giving rise to multiple Jacob isoforms with apparently different molecular weights.

Analysis of the primary structure of Jacob revealed a putative N-terminal myristoylation site and several potential phosphorylation sites for protein kinase C (PKC), cAMP-/cGMP-dependent protein kinases, and protein tyrosine kinases (Figures 1 and S1A). In addition, Jacob harbours a well-conserved bipartite nuclear localization signal (NLS). Interestingly, this NLS is part of an incomplete IQ motif (Figures 1 and S1A), a protein–protein interaction region characteristic for CaM binding [20–21].

### Jacob, a Synaptic and a Nuclear Protein

**Figure 2:**
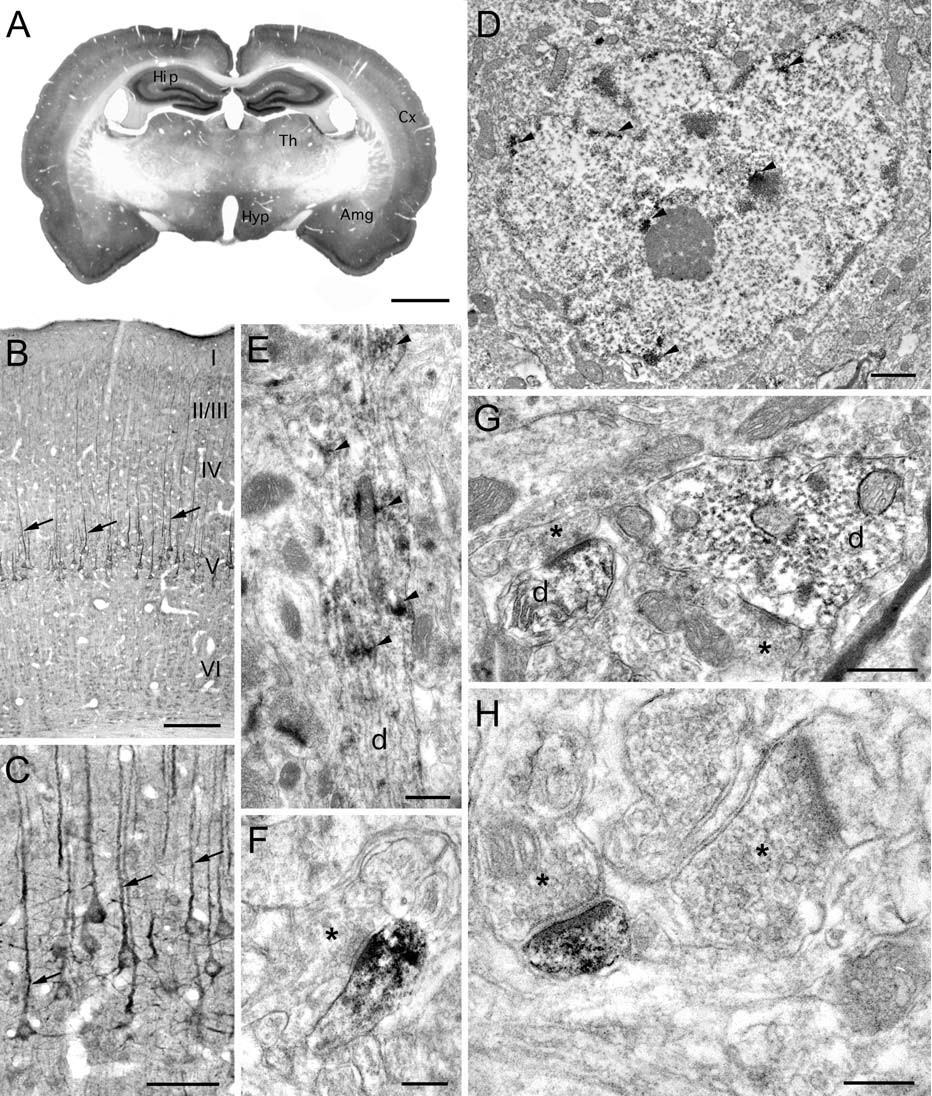
Immunolocalization of Jacob in Rat Brain (A) Low-magnification photograph of a rat brain coronal section through the forebrain (corresponding to Figure 31 in the rat stereotaxic atlas by Paxinos and Watson [64]) showing the distribution of Jacob IR. Immunostaining is particularly prominent in the hippocampus (Hip), the cerebral cortex (Cx), amygdala (Amg), and in nuclei of thalamus (Th) and hypothalamus (Hyp). (B and C) Apart from a fine neuropil staining, soma and apical dendrites (arrows) of pyramidal cells in the layer V of the cerebral cortex are strongly labelled. (D–H) Electron micrographs of the parietal cortex. (D) At the ultrastructural level, a patch-like distribution of the reaction product (arrowheads) is detectable in nuclei near the nuclear envelope and throughout the karyoplasm of cortical neurons. (E) Also in dendrites (d) of pyramidal cells, Jacob IR is not evenly distributed. Arrowheads mark patches of the immunoproduct. (G) Two profiles of immunopositive dendrites (d), which receive synaptic inputs (presynapses are marked by asterisks). In the larger dendrite, the reaction product is not concentrated towards the synaptic structures. (F and H) Examples of Jacob immunopositive spine synapses with labelled postsynaptic elements (in [H] in close proximity to an immunonegative spine synapse) that occur relatively rarely. Asterisks indicate presynaptic boutons. Preabsorption of the antibody with Jacob fusion protein blocked all immunostaining (unpublished data). Scale bars indicate 2 mm (A), 200 μm (B), 50 μm (C), 0.5 μm (D), 1 μm (E), and 0.25 μm (F–H).

In situ hybridization experiments revealed a strikingly restricted localization of Jacob transcripts in the limbic brain and cortical areas (unpublished data), showing extensive overlap with Caldendrin mRNA expression (see [19]). In accordance with in situ hybridization data, Jacob immunoreactivity (IR) was found predominantly in cortex and limbic brain structures, including the amygdala, the thalamus, and the hippocampus (Figure 2A). At the cellular level, particularly intense immunostaining was observed in the somatodendritic compartment of pyramidal cells in cortex (Figure 2B and 2C) and hippocampus, which closely resembles that seen for Caldendrin [16,19]. Moreover, both proteins extensively colocalize in hippocampal primary neurons (Figure S2).

**Figure 3:**
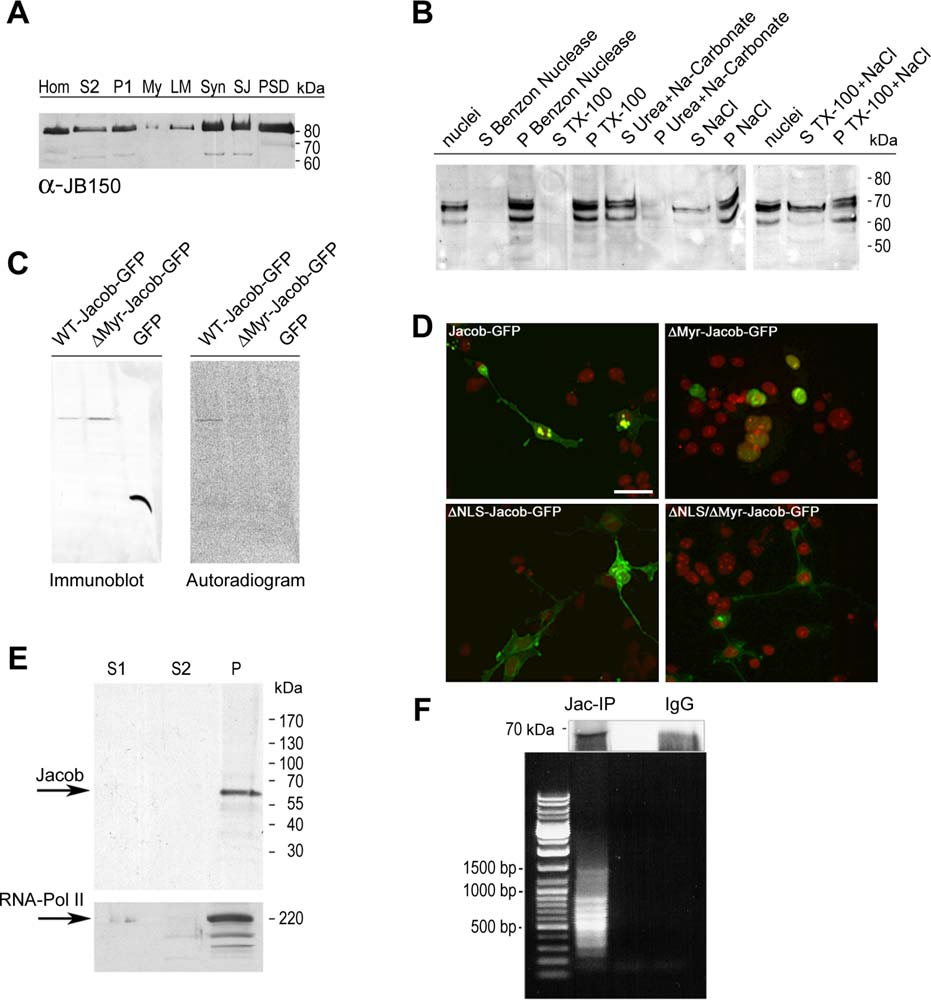
Jacob's Association with Subcellular Structures (A) Distribution of Jacob IR in subcellular fractions from rat brain. Panels show two different immunoblots of subcellular fractions (20 μg/lane) obtained by differential centrifugation from brains of adult rats visualized with Jacob (upper panel) and SAP90/PSD-95 (lower panel) antibodies. Hom, homogenate; LM, light membranes; My, myelin fraction; P2, crude membranes; PSD, postsynaptic density fraction; S2, 13.000 × *g* supernatant after removal of cell debris and nuclei; SJ, synaptic junctions; Syn, synaptosomes. (B) Western blots showing the extraction of Jacob from a nuclear protein-enriched fraction; P, remaining pellet after centrifugation; S, extractable supernatant. Nuclei were extracted with the agents indicated. Please note the tight association of Jacob isoforms with the nuclear protein fraction. Same amounts of protein were loaded. The size of marker proteins in kDa is indicated at the right margin. (C) Fractionation of chromatin after partial digestion of nuclei by micrococcal nuclease. P: heterogeneously sized DNA bound to the nuclear scaffold; S1: protein fraction containing mononucleosomal-sized DNA fragments; S2: protein fraction containing a nucleosomal ladder of DNA fragments. Jacob is only present in this fraction, which contains RNA Polymerase II and represents the euchromatin. (D) Chromatin immunoprecipitation of Jacob (Jac-IP) (immunoblot in the upper panel) using the P fraction as starting material leads to the coprecipitation of heterogeneously sized DNA (agarose gel in the lower panel) that is not present in IgG control (IgG). (E) N-myristoylation was examined in *wild-type Jacob (WT-Jacob-GFP)*, myristoylation mutant (*DMyr-Jacob-GFP*), and GFP-expressing HEK-293 (GFP) cells, which were incubated overnight with [^3^H] myristic acid. Crude detergent extracts were immunoprecipitated with a polyclonal GFP antibody, subjected to SDS-PAGE, transferred to nitrocellulose, and applied to a PhosphoImager system. Left: immunodetection with a monoclonal GFP antibody; right: autoradiograph. Note the incorporation of radiolabelled myristate into immunoprecipitated *WT-Jacob-GFP.* Sizes of marker proteins in kDa are indicated at the right margin. (F) Subcellular localization of *wild-type (Jacob-GFP)*, myristoylation mutant (*DMyr-Jacob-GFP*), NLS mutant (*DNLS-Jacob-GFP*), and myristoylation-NLS double mutant (*DNLS/DMyr-Jacob-GFP*) Jacob GFP-chimeras expressed in COS-7 cells for 24 h. For counterstaining of nuclei, cells were embedded in propidium iodide–containing mounting media (red). Very little variability was seen in the subcellular localization of these different constructs (*DMyr-Jacob-GFP* in 98% of all cases nuclear, whereas *DNLS-Jacob-GFP* and *DNLS/DMyr-Jacob-GFP* were in 98% of all cases extranuclear). Scale bar indicates 25 mm.

At the ultrastructural level, Jacob IR was localized to a subset of asymmetric type I synapses on dendrites of cortical neurons (Figure 2F–2H). Apart from its synaptic localization, intense label was present in patches in dendrites (Figure 2E). In these patches, Jacob IR was mainly concentrated at the cortical cytoskeleton. In contrast to Caldendrin, intense Jacob immunolabelling was also seen in neuronal nuclei (Figure 2D). Patchy IR was found both at the nuclear envelope and in the nuclear matrix. Subcellular fractionation experiments confirmed that Jacob is a synaptic and a nuclear protein. Differential centrifugation of brain protein fractions demonstrated that Jacob IR is associated with particulate fractions, including light and heavy membranes, and is prominently present in synaptosomes, synaptic junctional membranes, and the PSD fraction (Figure 3A). Jacob, like Caldendrin [16], is tightly associated with the PSD, since extensive Triton X-100 extraction resulted in a further relative enrichment of Jacob IR in the detergent-extracted PSD fraction. Interestingly, immunoblots of the crude nuclear fraction demonstrated the presence of prominent Jacob IR bands in the range of 62–70 kDa, whereas the major bands detected in PSD preparations migrated at 72–80 kDa in SDS-PAGE (Figure 3A and 3B), a difference that most likely reflects posttranslational modification(s).

The architecture of the nucleus includes two overlapping nucleic acid–containing structures that are directly associated with the regulation of gene expression: the chromatin and the nuclear matrix. Therefore, we isolated highly pure nuclear matrix, heterochromatin, and euchromatin fractions. Interestingly, after chromatin fractionation, Jacob was found to be exclusively associated with the RNA polymerase II–containing euchromatin (Figure 3C). Moreover, the Jacob-containing protein complexes immunopurified from euchromatin also contained significant amounts of DNA (Figure 3D). In addition, the initial purification of the crude nuclear fraction showed an enrichment of the 62–70 kDa Jacob bands in the nuclear matrix (D. C. Dieterich and M. R. Kreutz, unpublished data). This subcellular localization could be confirmed by subsequent extraction of nuclear protein components, including chromatin from COS-7 cells transfected with a green fluorescent (GFP)-Jacob fusion protein (D. C. Dieterich and M. R. Kreutz, unpublished data). These findings suggest that Jacob is highly enriched at active sites of nuclear gene transcription and mRNA processing.

### N-myristoylation of Jacob Is a Prerequisite for Its Extranuclear Localization

Jacob harbours an N-terminal myristoylation consensus motif. Transfection of HEK-293 cells cultivated in the presence of ^3^H-myristic acid with a *wild-type* (*WT*)-*Jacob-GFP* construct, subsequent immunoprecipitation with a monoclonal anti-GFP antibody and immunoblotting revealed incorporation of radioactivity at a band immunoreactive to both a polyclonal GFP antibody (Figure 3E) and Jacob antiserum (unpublished data). No incorporation was seen in controls transfected with GFP alone or with a myristoylation mutant, Δ*Myr-Jacob-GFP*, in which the crucial glycine at position 2 was mutated to alanine (Figure 3E). In contrast to the *WT-Jacob-GFP* construct, transient transfection of COS-7 cells with the Δ*Myr-Jacob-GFP* construct led to an exclusive nuclear localization of the mutant protein (Figure 3F).

Jacob's primary structure exhibits a well-conserved bipartite NLS between aa 250–265. To test for the functionality of this NLS, we generated a deletion mutant (Δ*NLS-Jacob-GFP*) lacking the six basic amino acid residues between 247–252. Transfection of this construct in COS-7 cells resulted in an extranuclear localization of the mutant protein (Figure 3F). Hence, the bipartite NLS seems to be necessary and sufficient for nuclear import of Jacob as the double mutant Δ*NLS/*Δ*Myr-Jacob-GFP* is extranuclear in transfected COS-7 cells.

### Nuclear Jacob Accumulation Results in a Rapid Stripping of Synaptic Contacts and a Simplification of Dendritic Processes

**Figure 4:**
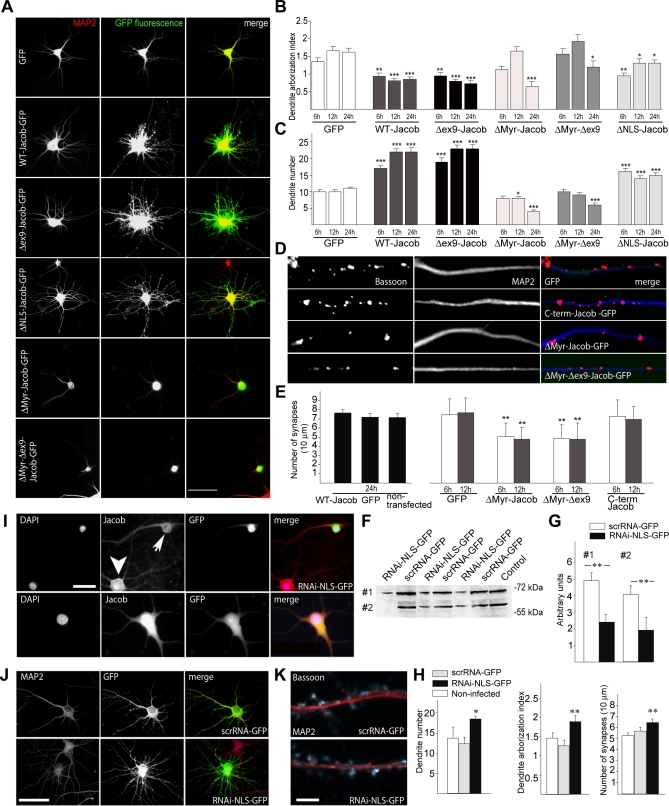
Morphological Analysis of Primary Neurons after Jacob Overexpression or RNAi Knockdown (A) Micrographs depicting MAP2/GFP-labeled hippocampal neurons 24 h after transfection with different Jacob constructs. Neurons were transfected at DIV11. Scale bar indicate 30 μm. (B) Quantification of dendritic complexity by determination of the average number of dendritic branch points for each MAP2-immunopositive dendrite (dendrite arborization index/N: 70 cells in each group). An asterisk (*) indicates *p* < 0.05; double asterisks (**) indicates *p* < 0.01; and triple asterisks (***) indicates *p* < 0.001. (C) Quantification of MAP2-immunopositive neurites after overexpression of different Jacob-GFP cDNA constructs and GFP-vector as a control (*n* = 70 cells in each group). An asterisk (*) indicates *p* < 0.05; triple asterisks (***) indicates, *p* < 0.001. (D) Micrographs depicting Bassoon-immunoreactive synaptic puncta on dendrites of hippocampal primary neurons after overexpression of different Jacob cDNA constructs. Scale bar indicates 2 μm. (E) Quantification of synaptic puncta on dendrites of hippocampal primary neurons after overexpression of different Jacob-GFP cDNA constructs. Double asterisks (**) indicate *p* < 0.01. (F) Immunoblots demonstrating the knockdown of nuclear Jacob isoforms after lentiviral infection of cortical primary neurons. Immunoreactivity is clearly reduced in nuclear-enriched fractions at DIV21 after infection of cultures at DIV0 with an RNAi virus targeted to knockdown specifically the NLS-bearing nuclear isoforms of Jacob (RNAi-NLS-GFP). A scrambled version of the targeting sequence (scrRNA-GFP), however, has no effect. (G) Immunoblot quantification of the knockdown of Jacob nuclear isoforms with the Quantity One software from BioRad. Depicted are three independent experiments. Quantitative immunoblot analysis was done for the upper (#1) and lower band (#2) (*n* = 5). RNAi-NLS-GFP, RNAi virus-targeted knockdown of NLS-bearing Jacob isoforms. Arbitrary units are presented as mean ± SEM. Double asterisks (**) indicate *p* < 0.01. (H) Quantification of dendrite number and complexity and the number of synapses in RNAi-NLS-GFP– and scrRNA-GFP–infected neurons (infection at DIV0/pictures taken at DIV21) as compared to noninfected controls. An asterisk (*) indicates *p* < 0.05; double asterisks (**) indicate *p* < 0.01. (I) Nuclear Jacob staining (red) is clearly reduced after infection of cortical primary neurons with the lentiviral RNAi-NLS-GFP construct (green; sharp arrow, upper panel) as compared to noninfected cells from the same culture (thick arrow, upper panel) or neurons infected with the scrRNA-GFP construct (lower panel). Blue channel: nuclear DAPI staining. Infection was done at DIV0, fixation at DIV21. Scale bar indicates 10 μm. (J) MAP2/GFP-labelled cortical primary neurons after infection with the lentiviral RNAi-NLS-GFP and the scrRNA-GFP construct (infection: DIV0/fixation: DIV21). Note the morphological phenotype of NLS-Jacob knockdown. Scale bar indicates 30 μm. (K) Bassoon-immunoreactive synaptic puncta (blue) on MAP2-stained (red) dendrites of cortical primary neurons after infection with the lentiviral RNAi-NLS-GFP and the scrRNA-GFP construct. (infection: DIV0/fixation: DIV21). Scale bar indicates 4 μm.

To elucidate functional consequences of nuclear versus extranuclear localization of Jacob in terms of structural plasticity, we transfected hippocampal primary neurons with different mutant (Δ*NLS-Jacob-GFP* or Δ*Myr-Jacob-GFP*) or *WT-Jacob* constructs. Transfection of these different mutants had drastic effects on cell morphology. *WT-Jacob-GFP*–transfected neurons as compared to GFP controls exhibited more, but less-complex, dendritic processes (Figures 4A–4C and S3A). This effect was astonishingly rapid and was observed already after 6–12 h post-transfection. In sharp contrast to the *WT-Jacob-GFP* overexpression phenotype, Δ*Myr-Jacob-GFP*–transfected cells lost most of their dendritic processes within 12 to 24 h (Figure 4A–4C). In these cells, the construct exclusively accumulated in the nucleus (Figure 4A). Interestingly, the density of synaptic puncta was already reduced before the retraction of dendrites became visible, observed as early as 6 h following transfection (Figure 4D and 4E). No effect on synapse number of *WT-Jacob-GFP* was seen even 24 h after transfection (Figure 4E). This strongly suggests that the simplification of the postsynaptic receptive units precedes the retraction of the dendrite. The opposite was found after a lentiviral RNA interference (RNAi)-based knockdown of the nuclear Jacob isoforms harbouring exon 6 with the NLS (*RNAi-NLS-GFP*). Quantitative immunoblot analysis and immunostainings showed that viral infection of cortical primary cultures led to a specific reduction of these nuclear Jacob isoforms (Figure 4F, 4G, and 4I). Infected cells showed a slightly increased number of synapses and a more complex dendritic cytoarchitecture (Figure 4H, 4J, and 4K). Similarly, overexpression of Δ*NLS-Jacob-GFP* caused an increase in the number of dendrites, but had no effects on the number of synapses (unpublished data), providing further evidence that the reduction of synaptic contacts directly correlates with the presence of Jacob in the nucleus.

Alternative splicing generates splice isoforms like Δ*ex9-Jacob* that contain the NLS but lack large parts of the carboxy-terminus (Figure S1B). We generated a myristoylation-deficient construct of this isoform (Δ*Myr-*Δ*ex9-Jacob-GFP*), which accumulated in the nucleus after transfection of primary neurons, and its overexpression resulted in a comparable reduction of dendritic complexity and synapse number as those seen with Δ*Myr-Jacob-GFP* (Figure 4A–4E). On the other hand, overexpression of a construct lacking the first 235 amino acids (*C-term-Jacob-GFP*) had no morphological consequences despite the presence of the NLS and its exclusive nuclear localization (Figures 4E and S3). Taken together, these data provide strong evidence that the reduction of synaptic contacts directly correlates with the presence of Jacob in the nucleus and that the N-terminal half and the NLS are pivotal for Jacob's morphogenetic impact on dendritic architecture.

### Caldendrin Binds the Region of Jacob That Harbours the Bipartite NLS in a Ca^2+^-Dependent Manner and the Binding Cannot Be Competed by CaM

**Figure 5:**
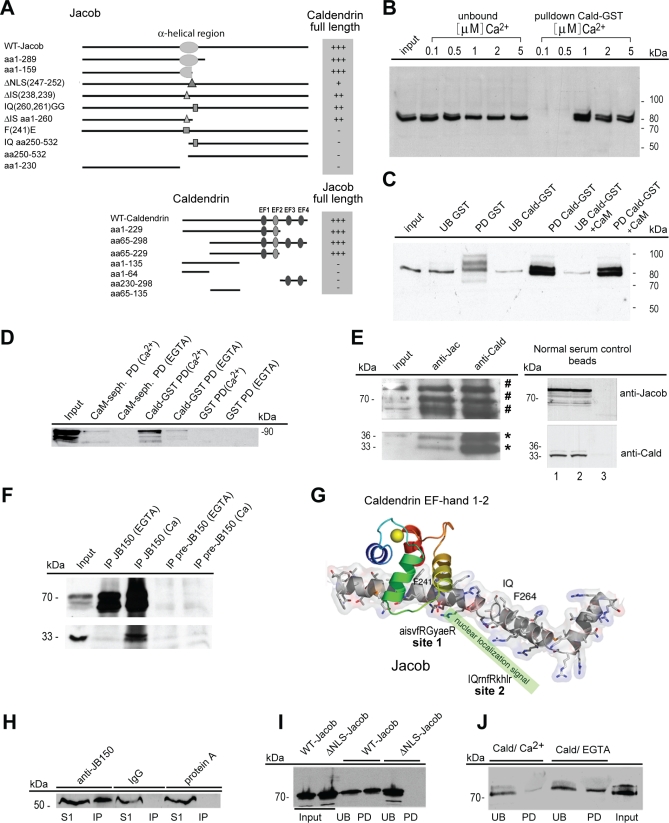
Caldendrin and Importin-a Bind to Jacob in a Competitive Manner (A) Mapping the Caldendrin–Jacob interaction using the yeast two-hybrid assay. Upper panel: deletion and point mutation constructs of Jacob tested for interaction with full-length Caldendrin. Lower panel: deletion constructs of Caldendrin tested for interaction with full-length Jacob. Yeast two-hybrid interactions were quantified based on the time course of induction of the reporter gene β-galactosidase. Triple plus signs (+++) indicate blue colonies after 1 h; double plus signs (++) indicate blue colonies after 3 h; a plus sign (+) indicates blue colonies after 6 h; a negative sign (–) indicates no signal after 6 h. (B) Interaction of Jacob with GST-tagged Caldendrin (Cald-GST) in a pull-down assay at different Ca^2+^ concentrations indicated. A Triton X-100–extracted P2 fraction was incubated with glutathione sepharose loaded with a GST fusion protein containing the C-terminal half (aa 137–298) of Caldendrin or with GST alone. pull-down, pellet fraction; unbound, supernatant. (C) Interaction of Jacob with GST-tagged Caldendrin in a competitive pull-down assay with recombinant CaM at 100 μM Ca^2+^. A Triton X-100–extracted P2 fraction was incubated with glutathione sepharose loaded with a GST-fusion protein containing the C-terminal half (aa 137–298) of Caldendrin or with GST alone in the presence or absence of 40-μg recombinant CaM. PD, pull-down fraction; UB, unbound material. After washing, bound proteins were detected by western blotting using the antibody anti-JB150. (D) WT-Jacob-GFP does not bind to CaM in a pull-down assay. Jacob binding to CaM or Caldendrin-GST sepharose was tested in the presence of 100 μM Ca^2+^ or 2 mM EGTA. GST sepharose was used as the control. WT-Jacob-GFP was detected by western blotting with a GFP antibody. (E) Co-immunoaffinity purification of Jacob and Caldendrin. Left panel: 15 mg of protein from the 100,000 × *g* supernatant of a rat brain membrane extract were loaded either to an anti-Jacob affinity column (anti-Jac) or to an anti-Caldendrin affinity column (anti-Cald). Jacob is co-eluted with Caldendrin and vice versa from the corresponding affinity columns. A number sign (#) indicates Jacob-immunoreactive bands and an asterisk (*) Caldendrin-immunoreactive bands. Right panel: no specific binding was seen to normal serum control beads. 1: Deoxycholic acid (DOC) extract from rat brain cortex; 2: nonbinding proteins (flow-through); 3: specifically bound proteins (eluate). (F) Caldendrin coimmunoprecipitates with Jacob from a rat brain extract in a Ca^2+^-dependent manner. Rabbit anti-Jacob bound to protein A sepharose specifically precipitates Jacob independent from the conditions used (5 mM of EGTA or 100 μM Ca^2+^ in the precipitation buffer). Rabbit IgG is used as a negative control (upper panel). Caldendrin was only detected in the precipitate under Ca^2+^ conditions (lower panel). (G) Molecular modelling of the Caldendrin–Jacob interaction. Caldendrin binds Ca^2+^-dependently to the first incomplete IQ motif, and blocks the NLS. As indicated, Jacob contains two incomplete IQ motifs. The matching residues are marked in bold. The helical structure has been modelled using the coordinates of Myosin I (pdb: 1wdc), showing that Jacob has a similar CaM site distribution as the template structure. In contrast to myosin I/CaM, Caldendrin binds to site 1 in a complex more closely related to the compact CaMKII/CaM complex. This is in agreement with Caldendrin's binding at high Ca^2+^ concentration, whereas IQ motif proteins bind independently of Ca^2+^ concentrations. Crucial residues for the interaction are indicated. (H) Immunoprecipitation of Importin-α1 from a soluble rat brain protein fraction with a Protein-A sepharose-coupled Jacob antibody (JB-150). Importin-α1 was only found in the immunoprecipitate (IP) of the Protein-A sepharose coupled Jacob-antibody, whereas it remained in the prewashing supernatant (S1) in the IgG and Protein-A sepharose control. (I) GST-Importin-α1 pull-down of myc/his-tagged WT and DNLS-Jacob extracted from transfected HEK-293 cells. Only WT Jacob, but not DNLS-Jacob, is found in the pull-down, indicating that the presence of the NLS is essential for the Importin-α1/Jacob interaction. PD, pull-down fraction; UB, unbound material. (J) GST-Importin-α1 pull-down of myc/his-tagged WT Jacob in the presence of equimolar amounts of Caldendrin. Pull-down of Jacob is attenuated in the presence of 2 mM Ca^2+^, but not in the presence of 2 mM EGTA. PD, pull-down fraction; UB, unbound material.

Since the NLS is not only essential, but also sufficient to target Jacob fragments to the nucleus, and the N-terminal half of the protein is crucial and sufficient to elicit the strong pleiomorphic negative effects on neurite and synapse number of nuclear Jacob, we next investigated the Caldendrin binding region in Jacob and vice versa, as well as the functional consequences of Caldendrin binding in more detail. Mapping of binding domains within both proteins was performed using deletion constructs for cotransformation in yeast two-hybrid assays. In Caldendrin, the region containing the first and the second, probably cryptic, EF-hand was found to be essential for Jacob binding (Figure 5A). Strikingly, in Jacob, we could map the Caldendrin binding region to the central α-helical region that harbours the bipartite NLS. Deletion of the first six basic residues of the NLS led to significantly reduced Caldendrin binding (Figure 5A).

To substantiate the yeast two-hybrid data, we verified the interaction between Jacob and Caldendrin, employing pull-down assays from brain tissue using a glutathione S-transferase (GST)-Caldendrin fusion protein. In this assay, the interaction of Caldendrin and Jacob was found to be Ca^2+^ dependent inasmuch as 1 μM free Ca^2+^ in the buffer was required to pull down recombinant Jacob (Figure 5B). Strikingly, CaM did not bind to Jacob at any Ca^2+^ concentration tested (Figure 5C), and CaM did not compete with GST-Caldendrin for binding to Jacob (Figure 5D). Further evidence for a bona fide interaction of the two proteins was provided by the binding of Jacob to an anti-Caldendrin antibody column and vice versa (Figure 5E). These findings are consistent with the colocalization of Jacob and Caldendrin in dendrites and dendritic spines in hippocampal primary neurons observed by confocal laser scans (Figure S2B). Importantly, the coimmunoprecipitation of Caldendrin from rat brain required the presence of Ca^2+^ and was not visible after addition of EGTA to the precipitation buffer (Figure 5F). Thus, the interaction of Caldendrin and Jacob in vitro and in vivo is strictly Ca^2+^ dependent.

Three-dimensional modelling substantiated the idea that structural differences in Jacob binding surfaces of Caldendrin and CaM [18] can explain the above findings. In crystallographic structures of CaM-peptide complexes, a helical peptide binds to one or both hydrophobic pockets formed by pairs of either EF-hands 1 and 2 or EF-hands 3 and 4 of CaM. The residues forming the two hydrophobic binding pockets are identical in CaM and Caldendrin. However, the binding affinities for the various peptides are given by the size and shape of the pocket, which has been classified as open, semi-open, or closed [22]. This size is dynamically regulated by the Ca^2+^ binding states of the EF-hands, which are not rigid structural units, but may function as hinges at low Ca^2+^ concentration [23]. Indeed, the second EF-hand of Caldendrin is incapable of binding Ca^2+^, which in turn results in different binding dynamics at the first hydrophobic pocket compared to CaM.

Interestingly, the Jacob sequence does not match any pattern for a typical CaM binding site, but has two incomplete IQ motifs (residues 237–247, aisvf**RG**yae**R**, and residues 260–269, **IQ**rnf**R**khlr) within a central region (residues 229–272) containing the bipartite NLS (residues 247–266, PSORTII, [24]). Several CaM binding sequences within α-helices have been defined that contain an IQ motif [20–21,25], which was originally suggested to classify Ca^2+^-independent peptide binding according to the referential CaM/IQ complex. Modelling of the Caldendrin interaction site (Figure 5G) suggested that the phenylalanine at position 241 is absolutely essential for the protein interaction, and we therefore generated a point mutation at this position (substitution of F to E). This F241E mutant construct indeed showed no interaction with Caldendrin in yeast (Figure 5A), supporting the assumption that the first part of the central α-helix overlapping with the NLS is the Caldendrin binding region. This α-helical region fits neatly into the hydrophobic pocket generated by EF-hands 1 and 2 and resembles binding to the Ca^2+^ sensor protein in the manner of an “open” hydrophobic pocket as closely related to the binding of CaMKII to CaM, than the “semi-open” binding in MyosinI/CaM. Moreover, this largely excludes the second incomplete IQ motif as an active binding site for Caldendrin. Accordingly, mutations of residues 260 and 261, IQ to GG, yielded only minor differences in the binding properties of Jacob to Caldendrin in yeast (Figure 5A). In summary, it is predicted that Caldendrin will bind to comparable structures in the presence of a large excess of CaM, suggesting that the Caldendrin–Jacob interaction has evolved independently of CaM-signalling pathways. Even more interesting, binding of Caldendrin to Jacob can be predicted to reduce or inhibit the accessibility of the NLS.

### Importin-α Binding to Jacob's NLS Is Competed by Caldendrin

The transport of proteins from the cytosol through the nuclear pore complex into the nucleus depends on the binding of Importins to a specific NLS within the cargo. Within this scheme, Importin-α functions as an adapter molecule by binding both the NLS-bearing protein and Importin-β. Structural modelling suggests that Caldendrin binding will potentially occupy Jacob's NLS, thereby masking this binding site for interaction partners that are likely involved in Jacob's nuclear localization. We tested this hypothesis first by confirming an interaction of Jacob with Importin-α. Coimmunoprecipitation of Importin-α1 from rat cortex indeed suggests a potential in vivo interaction of both proteins (Figure 5H). In pull-down experiments, we found specific binding of myc-his–tagged WT Jacob, but not of the Δ*NLS-Jacob* mutant to GST-Importin-α1 (Figure 5I). The binding of GST-Importin-α1 was not affected by the presence or absence of Ca^2+^ (unpublished data). We next investigated whether the binding of Importin-α1 can be competed by equimolar amounts of recombinant Caldendrin. Indeed, these studies revealed a competition between Caldendrin and Importin-α1 for binding to Jacob in the presence of Ca^2+^ (Figure 5J). Interestingly, no competition was seen in the presence of EGTA, suggesting that elevated Ca^2+^ levels are needed for Caldendrin to mask the NLS in Jacob.

### Nuclear Trafficking of Jacob Is Induced by NMDA Receptor Activation and Requires Importin-α Binding

An elegant recent study established a role of the classical Importin-mediated nuclear import for synapse-to-nucleus communication [26]. In this study, translocation of Importin-α1 and -α2 from distal dendrites to the nucleus was observed requiring NMDA receptor activity. Under resting conditions, however, dendritic Importins are largely immobile. Potential cargos associated with this translocation are at present unknown. Since Jacob is localized both to synapses and the nucleus, and harbours a bipartite NLS, which is bound by Importin-α1 and masked in a Ca^2+^-dependent manner by Caldendrin, we initially tested whether increased NMDA receptor activity will alter the intracellular localization of Jacob. For this purpose, we stimulated hippocampal primary cultures with NMDA for 3 min and quantified for endogenous Jacob the IR fluorescence signal intensity of propidium iodide–counterstained neuronal nuclei. Jacob IR increased significantly in neuronal nuclei within 30 min after NMDA receptor activation, with highest levels after 2 h (Figure S4A). Nuclear Jacob IR returned to control levels within 4 h (Figure S4A). As previously reported [26], Importin-α1 accumulates in the nucleus in a similar time frame. Interestingly, no recruitment of Caldendrin to the nucleus was observed (unpublished data). Bath application of glutamate led to a significantly increased nuclear accumulation of Jacob IR within a comparable time frame to NMDA receptor activation (Figure S4B). This accumulation could be completely blocked by coincubation of the competitive NMDA receptor antagonist DL-APV (DL-2-amino-5-phosphonopentanoic acid), indicating that activation of NMDA receptors is crucial for glutamate-induced recruitment of Jacob to neuronal nuclei (Figure S4B). To exclude the possibility that stimulation of primary neurons alters the accessibility of the nuclear Jacob antigen to the antibody, we performed quantitative western blot analysis on neuronal nuclei from organotypic hippocampal slice cultures stimulated with the same protocol. These experiments showed a significant increase in intensity of the two major Jacob nuclear isoforms (62 kDa/70 kDa) 2 h after stimulation (Figure S4C and S4D). Moreover, application of anisomycin after NMDA receptor activation did not affect the increase of nuclear Jacob IR, indicating that a recruitment of already existing extranuclear protein underlies the increased Jacob levels in hippocampal nuclei, but not de novo protein synthesis (Figure S4C and S4D).

**Figure 6:**
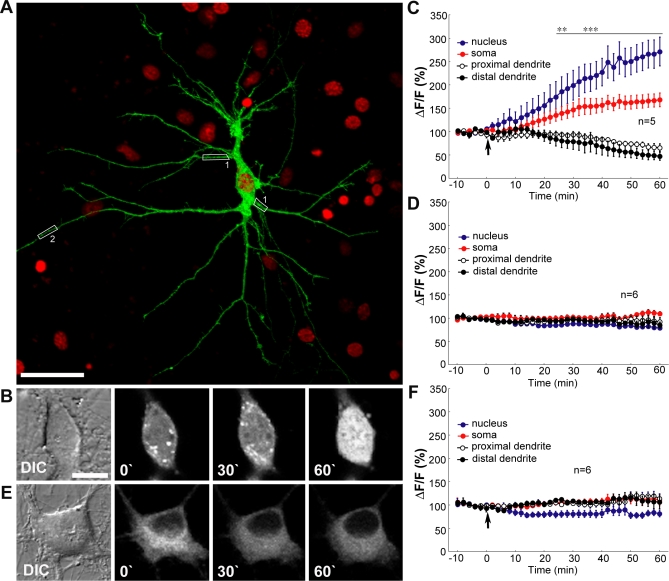
Time-Lapse Imaging of Jacob's Nuclear Translocation (A) Confocal maximal intensity image of a living hippocampal primary cultured neuron (DIV10) overexpressing WT-Jacob-GFP (green channel) and merged with a nuclear stain (DAPI) obtained after the experiment (red channel). (B) Representative selective video frames obtained from a WT-Jacob-GFP overexpressing neuron before (0) and after (from left to right) glutamate stimulation at time points indicated. DIC, difference interference contrast image of the neuronal soma. (C) Averaged temporal dynamics of the changes in WT-Jacob-GFP fluorescence intensity, quantified using ImageJ software in distinct regions of interest before and after stimulation with glutamate (50 μM). The arrow indicates the time point of glutamate application. The increase in WT-Jacob-GFP fluorescence in the soma and nuclei is accompanied by a reduction of fluorescence intensity in the dendrites. Statistically significant differences in GFP fluorescence in neuronal nuclei, somata and dendrites after stimulation in comparison to baseline fluorescence are indicated; double asterisks (**) indicate *p* < 0.01; and triple asterisks (***) indicate *p* < 0.001. (D) Without glutamate stimulation, no significant changes in *WT-Jacob-GFP* fluorescence in the nucleus as well as in soma and dendrites were observed. (E) Overexpression of the deletion mutant Δ*NLS-Jacob-GFP* leads to an extranuclear localisation of the mutant protein. Representative selective video frames obtained from Δ*NLS-Jacob-GFP* overexpressing neuron before (0) and after (from left to right) glutamate stimulation at time points indicated. DIC, difference interference contrast image of the neuronal soma. (F) Application of glutamate in Δ*NLS-Jacob-GFP*–transfected neurons does not change the GFP fluorescence levels in dendrites, soma, and nucleus. Scale bars indicate 40 μm in (A) and 25 μm in (B) and (E).

Using quantitative fluorescence time-lapse microscopy of hippocampal primary neurons transfected with *WT-Jacob-GFP* or the Δ*NLS* mutant, we found that the presence of the NLS is essential for the nuclear translocation of Jacob. Glutamate stimulation of *WT-Jacob-*GFP–transfected cultures kept in the presence of anisomycin resulted in an increase of somatic and nuclear GFP fluorescence with a time course comparable to that of the endogenous protein (Figure 6A–6D). The nuclear accumulation of Jacob-GFP, however, was not seen in neurons transfected with the Δ*NLS* mutant Jacob-GFP construct (Figure 6E and 6F), suggesting that the presence of the binding site for Importin-α1 is a prerequisite for Jacob's nuclear accumulation. Importantly, concomitant to the nuclear accumulation of *WT Jacob*, the GFP fluorescence decreased in proximal and distal dendrites (Figure 6C), an effect that was absent in Δ*NLS-Jacob-GFP*–transfected neurons. This indicates that the presence of the NLS and the interaction with Importin-α1 are not only important for the nuclear import, but are also crucial for Jacob's transport from dendrites to the nucleus.

### Activation of Largely Extrasynaptic NR2B-Containing NMDA Receptors Is the Most Efficient Stimulus for Jacob's Nuclear Import

**Figure 7:**
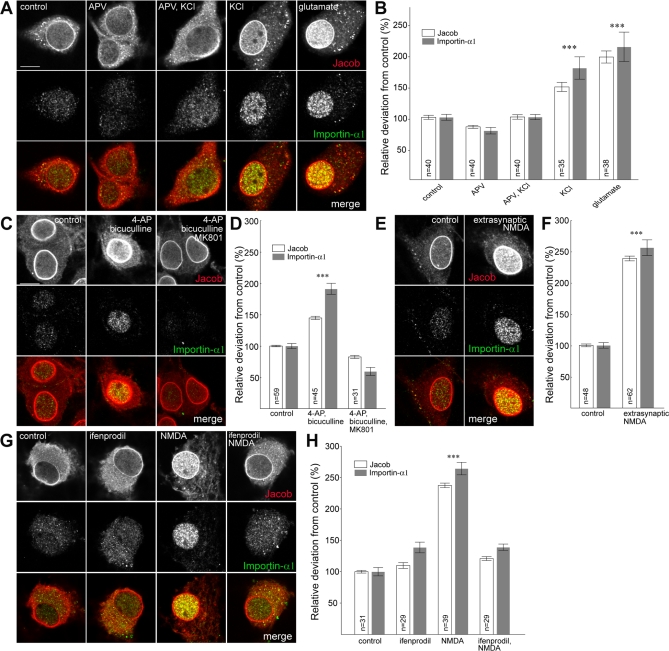
Nuclear Immunoreactivity of Jacob and Importin-α1 upon Different Stimulation Protocols Depicted are confocal images obtained from a single nuclear focal plane. Scale bar indicates 10 μm. All experiments were done in the presence of 7.5 μM anisomycin. Nuclear Jacob and Importin IR were quantified using the Image J software. The region of interest was outlined from DAPI stainings, and nuclear Jacob and Importin-α1 IR were measured as mean grey value (in arbitrary units of pixel intensity) from *Z*-stacks of two to three nuclear planes, and differences between groups were calculated as relative deviations from control. The nuclear membrane was excluded from the analysis. (A and B) Depolarization of hippocampal primary neurons (DIV16) for 3 min with 50 μM glutamate or 55 mM KCl in the presence or absence of the competitive NMDA receptor antagonist DL-APV (20 μM). Cultures were fixed 30 min after stimulation. Triple asterisks (***) indicate *p* < 0.001. (C and D) Synaptic stimulation of hippocampal primary neurons (DIV16) for 30 min with bicuculline (50 μM) and 4-AP (2.5 mM) in the presence or absence of the noncompetitive NMDA receptor antagonist MK801 (5 μM). Cultures were fixed 30 min after the stimulation with bicuculline started. Triple asterisks (***) indicate *p* < 0.001. (E and F) Extrasynaptic stimulation of hippocampal primary neurons (DIV16) with bath application of NMDA (100 μM for 3 min) following irreversible block of synaptic NMDA receptors. Cultures were fixed 30 min after NMDA stimulation. Triple asterisks (***) indicate *p* < 0.001. (G and H) Blockage of nuclear Jacob and Importin-α1 trafficking after bath application of NMDA (100 μM for 3 min) in the presence of the NR2B antagonist ifenprodil (5 μM). Cultures were fixed 30 min after NMDA stimulation. Triple asterisks (***) indicate *p* < 0.001.

To learn more about the role of Caldendrin for the extranuclear retention of Jacob, and to understand the apparently contradictory findings (i.e., NMDA receptor activation with subsequent Ca^2+^ influx leading to Jacob's nuclear import and concomitantly Caldendrin binding preventing this process at high synapto-dendritic Ca^2+^ levels), we analyzed the transport process of Jacob in more detail using confocal laser scan microscopy. A brief depolarization of hippocampal neurons with KCl also induced a translocation of Jacob and Importin-α1 to the nucleus in the presence of anisomycin (Figure 7A and 7B). However, the nuclear accumulation of both proteins was less pronounced than after glutamate receptor stimulation. Importantly, this effect was completely abolished in the presence of the NMDA receptor antagonist DL-APV (Figure 7A and 7B), indicating that raising intracellular Ca^2+^ levels by other means than NMDA receptor activation is not sufficient to drive Jacob and Importin-α1 into the nucleus.

NMDA receptors are situated both at synaptic and extrasynaptic sites [27,28]. Bath application of NMDA is considered to affect preferentially, but not exclusively, extrasynaptic NMDA receptors [6,7]. To differentiate between these two populations, we indirectly stimulated hippocampal cultures by incubation with the GABA_A_ receptor antagonist bicuculline. The blockade of inhibitory synapses leads to an increased release of glutamate at synaptic sites, and resulted as expected in an increased accumulation of Jacob and Importin-α1 in the nucleus (Figure 7C and 7D). This effect, however, was much less distinct as compared to the bath application of NMDA. A co-incubation with the noncompetitive NMDA receptor antagonist MK-801 attenuated the nuclear accumulation of Jacob and Importin-α1 to levels indistinguishable from control conditions (Figure 7C and 7D). Since MK-801 is an irreversible open channel blocker, we took advantage of this fact to differentiate between synaptic and extrasynaptic NMDA receptors. After washout of the drug following stimulation of synaptic glutamate receptors, we applied NMDA to the bath solution to exclusively activate extrasynaptic NMDA receptors. Interestingly, this regime induced a marked nuclear translocation of Jacob and Importin-α1 that was more prominent than the accumulation after stimulation of synaptic NMDA receptors (Figure 7E and 7F). Synaptic NMDA receptors contain predominantly the NR2A subunit, whereas their extrasynaptic counterparts contain mainly the NR2B subunit [29]. To test the hypothesis that the nuclear translocation of Jacob and Importin-α1 requires activation of NR2B-containing NMDA receptors, we repeated the experiments outlined above in the presence of the NR2B-specific antagonist ifenprodil. Intriguingly, in the presence of ifenprodil, the nuclear import of Jacob and Importin-α1 could be completely blocked after bath application of NMDA (Figure 7G and 7H). These results show that the nuclear import of these two proteins requires signalling via the largely extrasynaptically localized NR2B-containing NMDA receptors.

### Caldendrin Binding Targets Jacob Outside the Nucleus Only after Synaptic NMDA Receptor Stimulation

**Figure 8:**
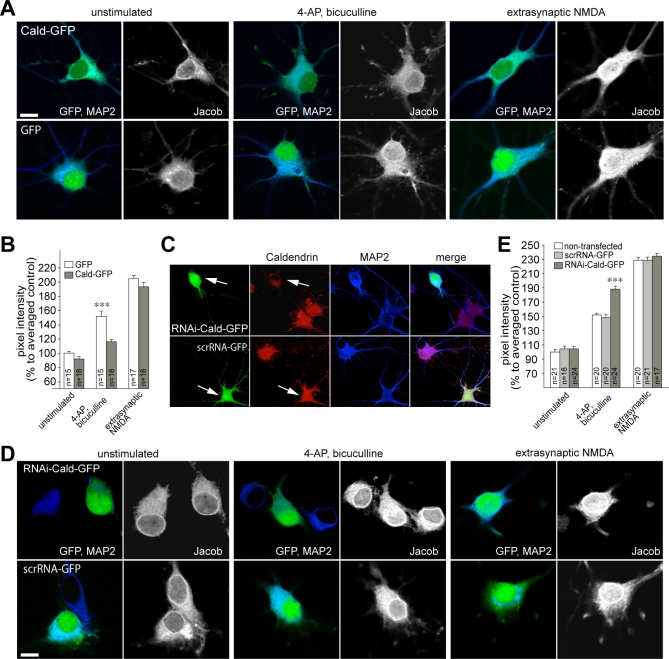
Caldendrin Targets Jacob Outside the Nucleus (A) Nuclear Jacob immunofluorescence following Caldendrin overexpression (upper panel) and in GFP control transfections (lower panel). Depicted are unstimulated neurons (first two rows), neurons 30 min after bicuculline stimulation (rows three and four), and neurons after stimulation of extrasynaptic NMDA receptors (rows five and six). Transfections were done at DIV13, stimulation experiments at DIV16. Scale bar indicates 10 μm (B) Quantitative analysis of nuclear Jacob immunofluorescence as percent deviation from unstimulated GFP control transfections. Triple asterisks (***) indicate *p* < 0.001. Data are presented as mean ± SEM. (C) Caldendrin staining (red) is clearly reduced after transfection of hippocampal primary neurons with a RNAi-GFP construct (GFP-positive cells are indicated with arrows, upper panel) as compared to nontransfected cells from the same culture or neurons infected with the scrRNA-GFP construct (see arrow in the lower panel). Blue channel: MAP2 staining. Transfection was done at DIV10, fixation at DIV16. Scale bar indicates 10 μm. (D) Synaptic and extrasynaptic stimulation of hippocampal primary neurons (DIV16). Cultures were transfected with a Caldendrin RNAi-GFP construct (upper panel) and the scrambled scrRNA-GFP construct (lower panel). Depicted are a transfected and a nontransfected neuron. Cultures were fixed immediately after the stimulation with bicuculline. Extrasynaptic stimulation of hippocampal primary neurons (DIV16) was done with the bath application of NMDA following the irreversible block of synaptic NMDA receptors. (E) Quantitative analysis of nuclear Jacob immunofluorescence after Caldendrin knockdown (RNAi-Cald-GFP), in scrambled controls (scrRNA-GFP) or non-transfected cells as percent deviation from unstimulated controls. Triple asterisks (***) indicate *p* < 0.001. Data are presented as mean ± SEM.

To follow up this hypothesis in more detail, we transfected a GFP-Caldendrin construct into hippocampal primary neurons. Expectedly, overexpression of Caldendrin blocked the increase of endogenous nuclear Jacob IR after synaptic stimulation at day in vitro (DIV) 16, indicating that the interaction with Caldendrin masks the bipartite NLS of Jacob (Figure 8A and 8B). However, after stimulation of extrasynaptic NMDA receptors, overexpression of Caldendrin attenuated Jacob's nuclear import much less efficiently (Figure 8A and 8B). We therefore checked whether RNAi knockdown of Caldendrin (Figure S5) affects the nuclear trafficking of Jacob differentially after synaptic and extrasynaptic NMDA receptor stimulation. We found that the nuclear immunofluorescence for Jacob was significantly increased in cells with reduced Caldendrin levels after enhancing synaptic activity with bicuculline at DIV 16 (Figure 8C–8E), whereas the Caldendrin knockdown had no effect on Jacob's nuclear import after activation of extrasynaptic NMDA receptors. This points to a regulatory function of this protein–protein interaction in nuclear trafficking of Jacob after enhanced synaptic activation that is related to the competitive accessibility of Jacob's NLS for either Caldendrin or Importin-α binding.

### Jacob Is Part of the CREB Shut-Off Pathway

**Figure 9:**
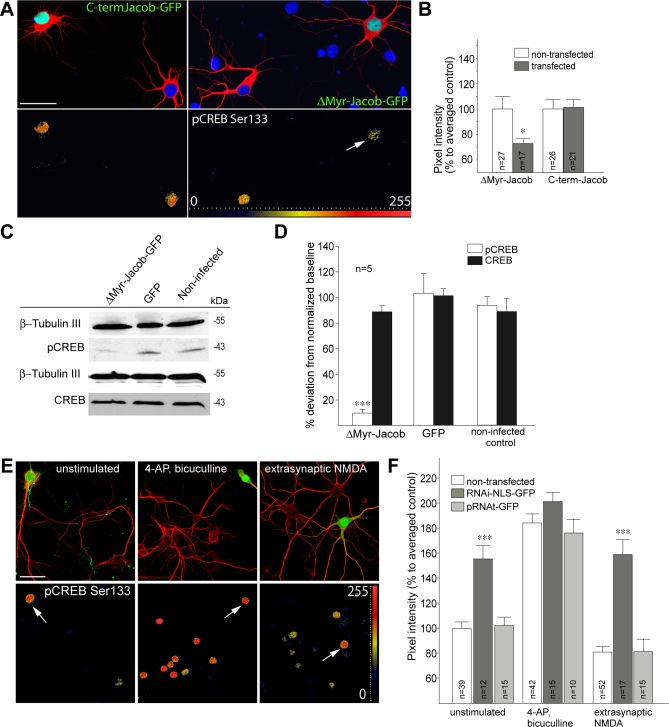
Nuclear Jacob Regulates the Phosphorylation of CREB Primary hippocampal neurons were transfected at DIV11, fixed the next day, and stained with MAP2-specific antibodies (Cy5), pCREB (Ser133; Alexa568), and mounted with DAPI-containing (blue) medium. Gradient lookup tables applied to determine the dynamic range of pCREB are included to visualize pixel intensity differences as indicated with the scale from 0 to 255. Scale bar indicates 40 μm. (A and B) Overexpression of Δ*Myr-Jacob-GFP* decreases the basal level of CREB phosphorylation in nonstimulated primary hippocampal cultures (DIV12). No effect as compared to nontransfected control neurons was seen after the transfection of a Jacob-GFP construct encoding the C-terminal half of Jacob. Quantification is shown in (B). A single asterisk (*) indicates *p* < 0.01. (C and D) Overexpression of Δ*Myr-Jacob-EGFP* using a Semliki Forest virus vector significantly reduced the level of pCREB at resting conditions in comparison to EGFP-infected and noninfected cortical primary neurons. The diagram (D) represents the data from four to five independent experiments normalized to β-Tubulin III. Total CREB levels were not affected by infection of the cultures. (E and F) Knockdown of nuclear Jacob utilizing the construct RNAi-NLS-GFP increases basal pCREB levels and prevents CREB shut-off after stimulation of extrasynaptic NMDA receptors. Prior to bath application of NMDA, cultures were stimulated with bicuculline in the presence of MK801. Arrows indicate RNAi-NLS-GFP–transfected neurons. Cultures were fixed 30 min after the stimulation with either bicuculline or the subsequent bath application with NMDA. Triple asterisks (***) indicate *p* < 0.001.

**Figure 10:**
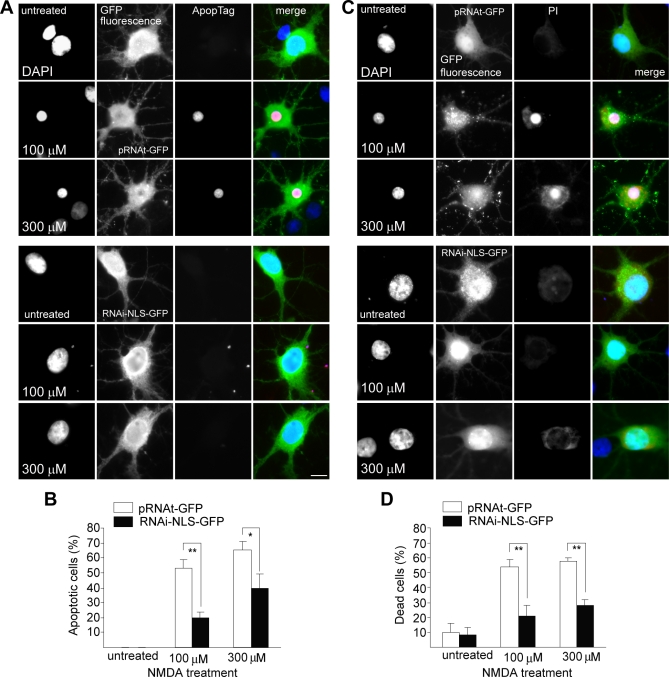
Nuclear Knockdown of Jacob Prevents NMDA-Induced Cell Death Primary hippocampal neurons transfected with the construct RNAi-NLS-GFP (DIV8) or with the corresponding pRNAt-GFP scrambled control vector were treated with 100 μM NMDA for 3 min or with 300 μM NMDA for 5 min at DIV15. Cell viability was assessed by fluorescence microscopy after nuclear staining with DAPI (blue in merge). Either incorporation of digoxigenin dNTPs as detected by using anti-digoxigenin–conjugated rhodamine antibody staining (red in merge; [A and B]) was used to quantify apoptosis (ApopTag), or propidium iodide staining (PI; red in merge; [C and D]) were employed as readouts indicating cellular degeneration. Cell viability was determined as the ratio of dead cells to the total number of transfected neurons. A single asterisk (*) indicates *p* < 0.1; double asterisks (**) indicate *p* < 0.01.

The predominant Ca^2+^- and NMDA receptor–activated signalling pathways to the nucleus in neurons funnel through the activation of the transcription factor CREB [1–2]. Previous work has shown that extrasynaptic NMDA receptor activation results in a dephosphorylation of CREB at Ser133 (pCREB) that renders it transcriptionally inactive and, therefore, constitutes a CREB shut-off signal [7,30]. Because Jacob was most efficiently targeted to neuronal nuclei after extrasynaptic NMDA receptor activation, we next addressed the question of whether the presence or absence of Jacob in the nucleus affects the phosphorylation of CREB at this crucial serine residue. As a first proof of principle, we explored whether nuclear overexpression of the Δ*Myr-Jacob-GFP* construct significantly reduced the levels of pCREB in hippocampal primary neurons as compared to untransfected or GFP-transfected controls under resting conditions (Figure 9A and 9B). Indeed, infection of cortical primary cultures with a Semliki Forest virus–expressing Δ*Myr-Jacob-GFP* led to drastically reduced pCREB levels as evidenced by quantitative immunoblotting, whereas total CREB levels were not affected (Figure 9C and 9D). To more rigorously test the hypothesis that Jacob is part of the CREB shut-off signalling pathway, we induced a knockdown of nuclear Jacob using plasmid-based RNAi constructs targeting exon 6–containing isoforms of the protein and subsequently stimulated extrasynaptic NMDA receptors with the protocol outlined above. We found that nuclear knockdown of Jacob completely abolished the reduction of pCREB observed after stimulation of extrasynaptic NMDA receptors (Figure 9E and 9F). These data point to a critical role of Jacob for survival of hippocampal primary neurons after triggering the CREB shut-off pathway. We therefore decided to assess next whether the absence of Jacob in the nucleus enhances neuronal survival after triggering CREB shut-off with the stimulation of extrasynaptic NMDA receptors. To this end, we chose in situ TdT-3′end labelling to visualize DNA fragmentation in hippocampal primary neurons as a measure of apoptotic cell death. Using this assay, we found that the number of neurons showing fragmented DNA after sustained extrasynaptic NMDA receptor activation was clearly reduced under conditions of nuclear knockdown of Jacob as compared to untransfected cells from the same plate or independent GFP-transfected controls from other plates (Figure 10A and 10B). Accordingly, the number of condensed propidium iodide–positive nuclei after nuclear knockdown of Jacob was reduced in the same manner as compared to controls (Figure 10C and 10D).

### Knockdown of Nuclear Jacob Prevents Stripping of Synaptic Contacts Induced by Sustained NMDA Receptor Activation

**Figure 11:**
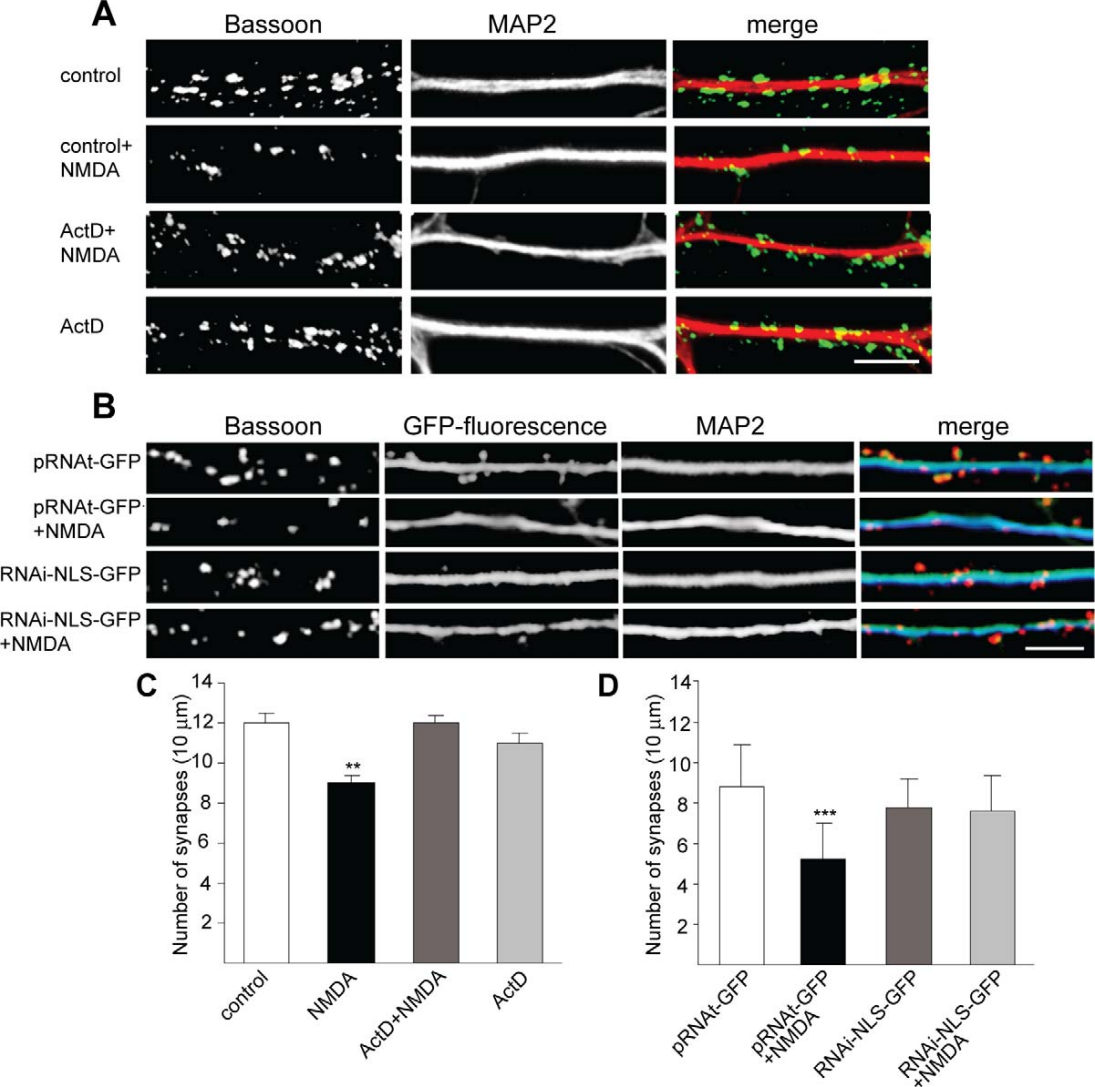
Nuclear Knockdown of Jacob Prevents NMDA-Induced Stripping of Synaptic Contacts (A and C) Untransfected primary hippocampal neurons (DIV13) were treated with 50 mM NMDA (bath application for 3 min), actinomycin D (ActD), or both. Cultures were fixed 4 h later and immunostained with anti-MAP2 and Bassoon antibodies to visualize dendritic processes (red in merge) and synapses (green in merge). Scale bar indicates 5 μm (A). Synapse density was quantified in (C). Double asterisks (**) indicate *p* < 0.01; triple asterisks (***) indicate *p* < 0.001. (B and D) Primary hippocampal neurons were transfected at DIV8 with a nuclear Jacob RNAi-NLS-GFP knockdown construct or scrambled pRNAt vector. At DIV13, cells were treated with NMDA or saline, and the number of synaptic puncta was quantified (D). Please note that transfection conditions are different than lentiviral infection in Figure 4H. At later time points than DIV13, the number of synapses increases after RNAi-NLS-GFP–based knockdown of Jacob as in Figure 4H. Scale bar indicates 5 μm. Double asterisks (**) indicate *p* < 0.01; triple asterisks (***) indicate *p*< 0.001.

A prominent consequence of bath application of NMDA to primary cultures is the reduction of synaptic contacts within a few hours [34]. Importantly, we found that this reduction requires gene transcription. Coincubation of NMDA with actinomycin-D, an inhibitor of RNA Polymerase II, completely blocked the loss of synaptic contact sites in treated cultures 4 h after stimulation (Figure 11A and 11C). Thus, in line with previous work, loss of synapses appears to be an early event of structural breakdown in cultures treated with bath application of NMDA and requires gene transcription. To further strengthen the point that Jacob is upstream of a transcription-dependent cell death pathway following excessive extrasynaptic NMDA receptor activation, we performed a plasmid-based RNAi knockdown of nuclear Jacob isoforms. Using this approach, we found that the knockdown of Jacob in the nucleus not only prevented CREB shut-off, but also preserved the structural integrity of transfected neurons. As evidenced by immunostainings with the presynaptic marker bassoon quantified 4 h after stimulation, the number of synapses in cultures transfected with RNAi targeting of nuclear Jacob isoforms was essentially the same compared to cultures transfected with a control RNAi vector (scrRNA-GFP). Most importantly, however, after bath application of NMDA, the number of synapses dropped only in cultures transfected with the control vector but not in Jacob RNAi-transfected cultures (Figure 11B and 11D). Thus, the absence of nuclear Jacob prevents not only CREB shut-off and subsequent neuronal degeneration, but also early events of structural disintegration related to loss of synaptic input.

## Discussion

### The Caldendrin–Jacob Interaction Constitutes a New Mechanism of NMDA Receptor-To-Nucleus Communication

**Figure 12:**
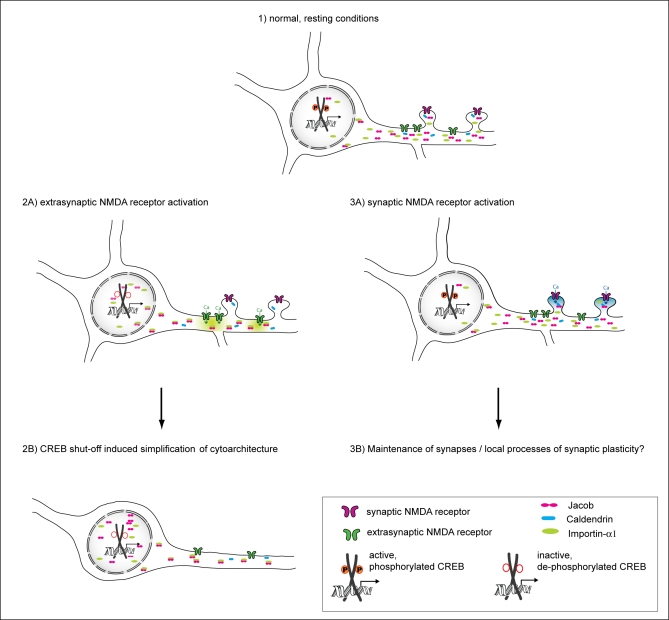
Model of Cellular Consequences of the Caldendrin-Jacob Pathway upon Extrasynaptic versus Synaptic NMDA Receptor Activation Under resting conditions (1), Jacob is mainly localized to the somatodendritic compartment in either an Importin-α-bound or –unbound state. Elevation of dendritic Ca^2+^ levels via activation of extrasynaptic NR2B-containing NMDA receptors (2A) causes Importin-α–bound Jacob to shuttle into the nucleus, inducing CREB shut-off and simplification of cytoarchitecture (2B). By contrast, activation of synaptic NMDA receptors gives rise to local high levels of Ca^2+^, enabling the binding of Caldendrin to Jacob, thereby accounting for its extranuclear localization (3A). Proposed consequences of this Ca^2+^-dependent Caldendrin–Jacob interaction may include maintenance of synapses and local modulations of synaptic plasticity (3B).

In this study, we identified a novel neuronal protein pathway that is well suited to couple NMDA receptor signalling to the cell nucleus and to trigger long-lasting changes in the cytoarchitecture of dendrites and the number of spine synapses. This novel pathway particularly couples activation of NR2B-containing NMDA receptors to morphogenetic signalling via the nuclear trafficking of Jacob. At resting conditions, Jacob is attached to extranuclear compartments in an either Importin-α bound or unbound state (see also Figure 12). Ca^2+^ influx through synaptic and extrasynaptic NMDA receptors is followed by a translocation of Importin-α from synapses and dendrites to the nucleus, and we propose that Importin-α–bound Jacob will be concomitantly recruited to the nucleus. Moreover, the presence of the NLS is essential for Jacob's translocation, indicating that trafficking from dendrites to the nucleus and not only nuclear import already requires the classical Importin pathway. This is reminiscent of previous data showing NMDA receptor-dependent Importin trafficking from dendrites to the nucleus [26], and establishes Jacob as the first identified cargo of this trafficking event. Accordingly, we always found a tight correlation between Jacob's and Importin-α1 nuclear translocation. Caldendrin binding can mask the bipartite NLS of Jacob in competition with Importin-α and thereby prevent its nuclear trafficking (Figure 12). However, in contrast to Importin-α binding, this requires high Ca^2+^ levels and not only NMDA receptor activation (Figure 12). We propose that Caldendrin will target Jacob to spine synapses after enhanced synaptic activation (Figure 12). In support of this hypothesis, we could provide evidence that activation of NR2B-containing NMDA receptors, which are mainly located at extrasynaptic sites, is crucial for the nuclear import of Jacob and Importin-α1. Interestingly, we found that blocking this receptor subtype did attenuate the nuclear accumulation of both proteins after stimulation of synaptic NMDA receptors that contain predominantly, but not exclusively, the NR2A subunit [29–31,35–38]. This suggests the intriguing possibility that the nuclear Jacob-Importin pathway is physically coupled to NR2B-containing NMDA receptors and that the presence or absence of Ca^2+^-bound Caldendrin in the respective synapto-dendritic compartment will decide whether local Jacob shuttles to the nucleus or not.

### Caldendrin and Jacob: A Protein Liaison in Control of the CREB Shut-Off Pathway

On the basis of the characteristics and consequences of its nuclear import, we found conclusive evidence that Jacob is part of the CREB shut-off pathway. The most prominent nuclear target of neuronal NMDA receptor signalling is the transcription factor CREB [1–2,8–9]. Subsequent to its phosphorylation at serine 133, pCREB triggers gene expression crucially involved in processes of synaptic plasticity and neuronal survival [8–9]. Analysis of this pathway has demonstrated that synaptic NMDA receptors strongly activate CREB-dependent gene expression, whereas extrasynaptic NMDA receptors trigger a CREB shut-off [7]. A most intriguing finding in recent years has been that the antagonistic signalling of extrasynaptic versus synaptic NMDA receptors resembles their opposing actions on the activation of ERK kinase [6,39–41]. Activation of synaptic NMDA receptors is coupled to the Ras-ERK pathway and subsequent CREB phosphorylation, whereas extrasynaptic NR2B-containing receptors promote dephosphorylation and inactivation of the Ras-ERK-pathway [6,39–41]. One caveat of this scenario, however, is that shutting down Ras-ERK alone cannot explain the shut-off of CREB since other mechanisms, and here prominently nuclear CaMK-IV, should be in principal sufficient to phosphorylate CREB in the absence of ERK activity [8–9]. Thus, the opposing influence of both types of NMDA receptors after bath application of NMDA requires another mechanism that will actively trigger CREB shut-off. Our data suggest that the same conditions that trigger shut-off of CREB and the Ras-ERK pathway drive Jacob into the nucleus. Overexpression of Jacob in the nucleus—without activating these pathways—is sufficient to attenuate CREB phosphorylation, and a nuclear knockdown of Jacob prevents CREB shut-off as well as neuronal cell death after triggering the pathway. Finally, the rapid loss of synaptic contacts, one of the hallmarks of bath application of NMDA in hippocampal primary cultures, was prevented by reducing the amount of nuclear Jacob. Noteworthy in this regard is the observation that CREB shut-off cannot be induced in young cultures (<DIV 7) [30,42], a developmental stage at which Jacob protein levels are very low (unpublished data). We therefore propose that nuclear Jacob is an essential component of CREB shut-off that might be actively involved in rendering CREB in a dephosphorylated state.

What is Jacob's physiological role in the nucleus? In initial experiments, we could not establish a direct binding of Jacob to CREB although both proteins are found in the overlapping fractions after gel filtration of nuclear protein complexes (unpublished data). Therefore, it is conceivable that Jacob is indirectly coupled via CREB-binding proteins to the CREB signalosome. To further support a role in gene expression, we provided substantial evidence that Jacob is highly enriched in two nuclear compartments associated with gene transcription and pre-mRNA processing. Jacob is abundant in euchromatin fractions and therefore present at active sites of gene transcription. The protein harbours long stretches of basic amino acid residues, which are well suited for DNA binding, although no known DNA binding motif was identified in its primary structure. Particularly with regard to the phenotype of its nuclear overexpression that involves a rapid destabilization of synaptic contacts and a retraction of dendrites, and which cannot be explained entirely by CREB shut-off, it is reasonable to assume that Jacob will be part of additional nuclear signalling events.

### Nuclear Jacob Induces Pleiomorphic Negative Effects on Synapto-Dendritic Cytoarchitecture

The nature of such signalling events will be obviously related to the circumstances of Jacob's nuclear trafficking. CREB shut-off has been largely assigned so far to pathophysiological insults, including spill-over of glutamate after excessive stimulation or reversal of glutamate transporters in the context of epileptic seizures or brain ischemia [3]. This view, however, probably has to be extended because in recent years, a number of observations raise the possibility that the activation of extrasynaptic NR2B-containing NMDA receptors can occur in a physiological context. It was shown that in several brain regions, sustained synaptic activation causes spillover of synaptically released glutamate to nonsynaptic sites [43–49]. In addition, sustained synaptic activation favours nonsynaptic release of glutamate from astrocytes [50–52], and it has been suggested that this glia-neuron transmission via extrasynaptic NMDA receptors has profound effects on non–Hebbian types of neuronal plasticity [53]. Moreover it was also claimed that activation of extrasynaptic NMDA receptors might directly induce heterosynaptic long-term depression at certain synapses in close proximity [54]. The evolving concept behind these studies is the idea of homeostatic scaling of synaptic input. Homeostatic plasticity refers to a process by which principal neurons in particular constantly adjust the integration of synaptic input to optimize the contribution of a single synapse with reference to its location in the dendrite and the synchronized activity in a given neuronal network [55,56]. A major aspect of homeostatic plasticity is the fact that uncontrolled potentiation of synapses will induce a ceiling effect characterized by epileptic activity and a decoupling of a given neuron from the dynamics of presynaptic input. Homeostatic plasticity reflects the necessity to either remove certain synapses that contribute less efficiently to the optimal activity within a neuronal network or to reduce the level of potentiation of synapses in this network. Jacob's nuclear accumulation and its rapid morphogenetic effects are in favour for a role in the regulation of plasticity-related gene expression related to homeostatic synaptic plasticity. Interestingly, this role includes a stripping of synaptic contacts that precedes the simplification and regression of dendritic processes. It is therefore conceivable that the loss of synapses is the initial trigger for the retraction of dendritic arbors. Moreover, this process is surprisingly rapid, indicating that synapses are actively destabilized. This in turn suggests that Jacob either blocks an essential nuclear signalling event required to prevent the removal of synaptic input or regulates the expression of genes that will actively destabilize synapses. It is likely that the CREB shut-off pathway will be part of this mechanism, but it is unclear whether it is sufficient to trigger solely the course of events following Jacob's nuclear import.

### Caldendrin and Jacob Provide a Novel Mechanism of Neuronal Ca^2+^ Signalling

A further intriguing aspect of this study is that it provides the first demonstration that an EF-hand CaM-like Ca^2+^ sensor protein regulates the nuclear localization of a protein by competitive binding to its NLS in a Ca^2+^-dependent manner. The significance of this novel mechanism of neuronal Ca^2+^ signalling is further underscored by the fact that binding of Caldendrin is specific in that its ancestor and closest relative in brain, CaM, did not bind to Jacob at any Ca^2+^ concentration tested. This is of importance since CaM levels are probably more than a magnitude higher in neurons than those of Caldendrin [18], and Ca^2+^ binding affinities are comparable between both proteins [57]. Computer modelling based on templates from crystallized structures shows that the outer surface of solvent-exposed amino acids, particularly EF-hand 2, which seems to be crucial for binding to Jacob, and another recently identified binding partner light chain 3 (LC3) [58] are covered by residues that clearly differ between CaM and Caldendrin [18]. Accordingly, LC3, a component of the microtubular cytoskeleton, apparently does not bind to CaM [58]. The principal specificity of Caldendrin protein interactions is further supported by the observation that very few mutations occurred in this region during vertebrate development and that none of these mutations affected the solvent-exposed amino acids of EF-hand 2 [18]. Thus, the singularity of the Caldendrin surface is intrinsic and independent from insertions or deletions, and we therefore suggest that this is probably due to adaptations of its surface to a specific localization and function in neurons of higher vertebrates.

How could this singularity with respect to other Ca^2+^ binding proteins relate to Caldendrin's neuronal function? In contrast to the interaction with Jacob, Caldendrin binding to most of its interaction partners is Ca^2+^ independent, as already described above for the LC3 interaction [57]. For instance, Ca^2+^-, CaM-, and ATP-independent interaction of the C-terminal half of Caldendrin/CaBP1 was demonstrated for the inositol trisphosphate receptor ((InsP_3_R) [59–60]. The functional consequence of Caldendrin binding is a reduction of InsP_3_-triggered intracellular Ca^2+^ release [59,60]. At the synapse, a Ca^2+^-independent binding was reported for L-type voltage-dependent Ca_V_1.2 Ca^2+^ channels [61,62]. This interaction will probably lead to increased Ca^2+^ currents following synaptic activation and thereby indirectly via increased synaptic activity could promote Caldendrin's and possibly Jacob's synaptic localization. Low synaptic activity and, hence, low synapto-dendritic Ca^2+^ levels will instead favour Caldendrin's binding to the InsP_3_R. It is therefore conceivable that Caldendrin can thereby directly lower Ca^2+^ levels in dendritic microdomains, and in consequence, negatively regulate its own association with Jacob. Therefore, a switch of binding partners could directly relate to Caldendrin's role in regulating Jacob's nuclear transition. Along these lines, it can be predicted that keeping the delicate balance between Jacob's nuclear and extranuclear localization via Caldendrin binding will provide a powerful regulatory mechanism in the transformation of dendritic Ca^2+^ signals into morphogenetic signals for the dendritic cytoarchitecture of principal neurons under pathophysiological and probably also under physiological conditions.

## Materials and Methods

### Yeast two-hybrid screening, cDNA cloning, and mRNA analysis.

Yeast two-hybrid screening was performed as described previously [63]. Library screening was done with a rat brain cDNA library in pACT2 (Matchmaker-GAL4 Two-Hybrid II; Clontech). The bait construct consisted of the entire open reading frame of Caldendrin cloned in frame into the pAS2–1 vector. A total of 3.5 × 10^6^ cotransformants were screened, and 108 clones were picked, which turned blue within 6 h in the initial test and after retransformation. Eight of these clones were found to encode a novel protein. Interactions were scored for ß-galactosidase activity by a colony lift assay. Binding activity of different constructs after retransformation was evaluated in three independent experiments.

A rat brain hippocampus cDNA Lambda ZAPII library (Stratagene) was screened with a cDNA probe encompassing the first 400 bp of Jacob's open reading frame. cDNA labelling, filter hybridization, and subcloning were done using standard procedures [16]. Cloning of full-length murine Jacob was done by reverse transcriptase PCR (RT-PCR) from mouse brain with primers encompassing the start and stop codon of rat Jacob. The PCR product was cloned into a TOPO TA vector (Invitrogen) and sequenced. A list of the constructs employed in this study is provided in Text S1.

### Generation of antisera.

Two peptides (aa 285–299 and aa 300–314), the GST fusion proteins GST-J_1–230_ and GST-J_253–404_ were used to immunize two rabbits and one guinea pig each. Specificity of the antibodies was tested on immunoblots of crude rat brain homogenate by preabsorption of the antibodies with corresponding N-terminal or C-terminal (J_262–532_) MBP fusion proteins or with affinity-purified antiserum.

### Immunostainings, time-lapse imaging, and quantification of neuronal morphology and immunocytochemistry.

Immunohistochemistry and immunocytochemistry were performed essentially as described previously [19] (see Text S1 for more details). Details of confocal laser scan microscopy and time-lapse imaging experiments can also be found under Supplementary Materials and Methods in Text S1.

### Western blot analysis, pull-down assays, immunoaffinity chromatography, and subcellular fractionation.

See Supplementary Materials and Methods in Text S1 for details.

### Lentiviral/plasmid-based sRNAi knockdown of Jacob and Caldendrin, Semliki Forest virus infection of cortical cultures.

For RNAi treatment, oligonucleotides with the sense/antisense sequence (19–21 bp) linked by a 9- or 10-bp–long stemloop sequence were obtained from Biomers. Sequences were as follows: nuclear Jacob knockdown (RNAi-NLS: 5′ AGA ATG ATT CCG CGT CTG TAA 3′/bp 892–912 of the Jacob cDNA); nuclear Jacob scrambled control (scrRNA: 5′ AGA TAT AGT CGC CGT CTG TAA 3′); all Jacob isoforms' knockdown (PAN-Jacob: 5′ TGC TAC TAG TTA CAG TGT AGA 3′/bp 390–410 of the Jacob cDNA); all Jacob isoforms' scrambled control (5′ TGA TAG GTC TAT ACG AGT TCA 3′), Caldendrin sRNAi (5′ TCC TGG CGG AGA CAG CAG ATA 3′/bp 665–685 of Caldendrin cDNA); and Caldendrin scrambled (5′ AGA ATC CTA AGA CAA GTG CAG 3′). Forward and reverse oligos were annealed, phosphorylated, and cloned BamHI, HindIII into the pRNAT-H1.1/Neo vector (Genscript) for plasmid-based RNAi knockdown. COS-7 cells were cotransfected with Flag-Jacob or Flag-Jacob-Myc/His and the RNAi expression vector. Cells were harvested 2 d after transfection and the samples solubilised for SDS-PAGE.

For lentiviral transfections, double-stranded, phosphorylated oligos were cloned BamHI/BglII, HindIII into the pZ-off vector and further subcloned EcoRI, AccI/BstBI into the FUGW H1(+) vector. HEK-293T cells were grown on polyD lysine-coated 10-cm^3^ plates to 90% confluence and cotransfected with the shRNA-FUGW H1(+) (10 μg), the VSVg (5 μg), and Δ8.9 (7.5 μg) vectors using Lipofectamine 2000 according to the manufacturer to produce competent virus particles. For virus production, cells were grown in Neurobasal medium supplemented with GlutaMAX and B27 at 32 °C and 5% CO_2_ overnight; the medium was changed and virus harvested 48–60 h after transfection. Sterile-filtered virus was directly added to primary cortical neurons at DIV0. After 3 wk, cells were either fixed with PFA for immunostaining or harvested and prepared for SDS-PAGE.

For the preparation of Semliki Forest particles and infection of primary cortical neurons, pSFV-Helper2, *pSFV-ΔMyr-Jacob-EGFP*, or *pSFV-EGFP* after in vitro transcription were cotransfected into CHO-K1 cells with Lipofectamine2000 (Invitrogen) according to the supplier's manual. After 24 and 48 h, the culture medium containing the budded particles was harvested. Viral particles were concentrated by ultracentrifugation through 10% sucrose, the pellet was resolved in Tris-buffered solution overnight at 4 °C. Aliquots of the particles were stored at −80 °C after shock freezing. For infection of primary cortical neurons, the particles were activated by chymotrypsin and further diluted with OptiMEM. High-density cortical cultures were infected at DIV16 and harvested 24 h later. Neurons were homogenized in 20 mM Tris buffer containing protease and phosphatase inhibitors, and solubilised in SDS buffer. The protein concentration was determined by amido black test, and equal amounts were loaded for SDS-PAGE.

### Statistical analysis.

Statistical analysis was performed with ANOVA and subsequent Bonferroni's Multiple Comparison test. Data are presented as mean ± standard error of the mean (SEM). A level of *p* < 0.05 was considered statistically significant.

### Computer modelling.

Details about the computer models can be found under Supplementary Materials and Methods in Text S1.

## Supporting Information

Figure S1 Primary Structure and Alternative Splicing of Jacob (1.5 MB TIF)

Figure S2 Jacob Associates with Dendritic Spines and Co-Localizes with Caldendrin (2.2 MB TIF)

Figure S3 The Nuclear Accumulation of Jacob's C-Terminal Half Has No Effect on Synapto-Dendritic Cytoarchitecture (898 KB TIF)

Figure S4 NMDA Induces the Nuclear Translocation of Jacob (360 KB TIF)

Figure S5 RNAi Knockdown of Jacob. (570 KB TIF)

Text S1 Supplementary Results and Materials and Methods (73 KB DOC)

### Accession Number

The Protein Data Bank (http://www.pdb.org/pdb/home/home.do) accession number for the structural model discussed in this paper is 1wdc.

## Abbreviations

aa: amino acid
CaM: calmodulin
CaMK: calmodulin kinase
CREB: cAMP response element-binding protein
DIV: day in vitro
ERK: extracellular signal-regulated kinase
GABA: gamma-aminobutyric acid
GFP: green fluorescent protein
GST: glutathione S-transferase
InsP_3_R: inositol trisphosphate receptor
IR: immunoreactivity
LC3: light chain 3
NCS: neuronal Ca^2+^ sensor
NLS: nuclear localization signal
PSD: postsynaptic density
RNAi: RNA interference
SEM: standard error of the mean
WT: wild type
