# Correction: Caldendrin–Jacob: A Protein Liaison That Couples NMDA Receptor Signalling to the Nucleus

**DOI:** 10.1371/journal.pbio.1000022

**Published:** 2009-01-27

**Authors:** Daniela C Dieterich, Anna Karpova, Marina Mikhaylova, Irina Zdobnova, Imbritt König, Marco Landwehr, Martin Kreutz, Karl-Heinz Smalla, Karin Richter, Peter Landgraf, Carsten Reissner, Tobias M Boeckers, Werner Zuschratter, Christina Spilker, Constanze I Seidenbecher, Craig C Garner, Eckart D Gundelfinger, Michael R Kreutz

Correction for:

Dieterich DC, Karpova A, Mikhaylova M, Zdobnova I, König I, et al. (2008) Caldendrin–Jacob: A protein liaison that couples NMDA receptor signalling to the nucleus. PLoS Biol 6(2): e34. doi:10.1371/journal.pbio.0060034

The version of Figure 3 originally published in this article was not the one intended for publication, and therefore does not correspond to the accompanying legend. The correct version of Figure 3 is published below.

**Figure 3:**
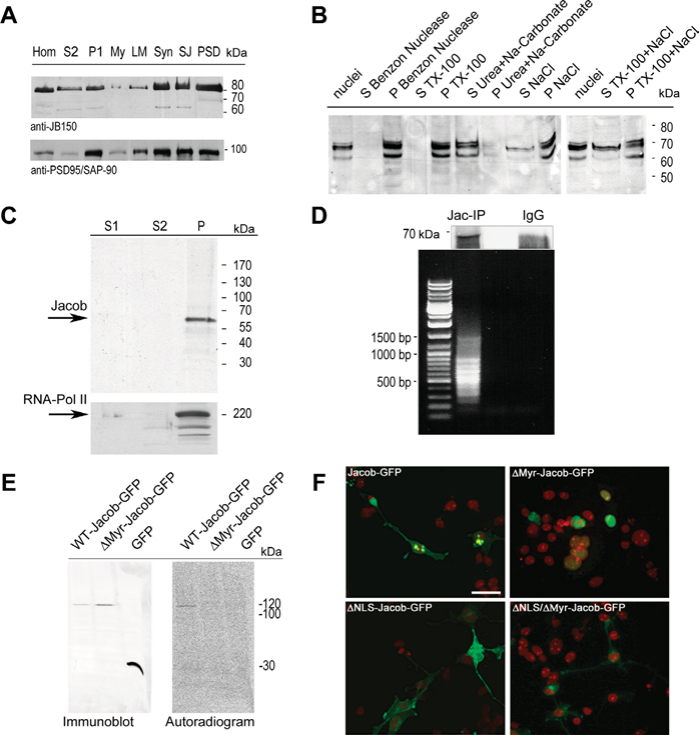
Jacob's Association with Subcellular Structures

Additionally, the authors would like to clarify the legend that accompanies Figure 3. The original final sentence is ambiguous, and should be replaced by “Jacob is only present in fraction P, which contains RNA Polymerase II and represents the euchromatin.”

