# Supplementary figures and images for: Caldendrin–Jacob: A Protein Liaison That Couples NMDA Receptor Signalling to the Nucleus
Source: PLoS Biol. 2008 Feb 26;6(2):e34. doi: 10.1371/journal.pbio.0060034 (PMC2253627; doi:10.1371/journal.pbio.0060034)

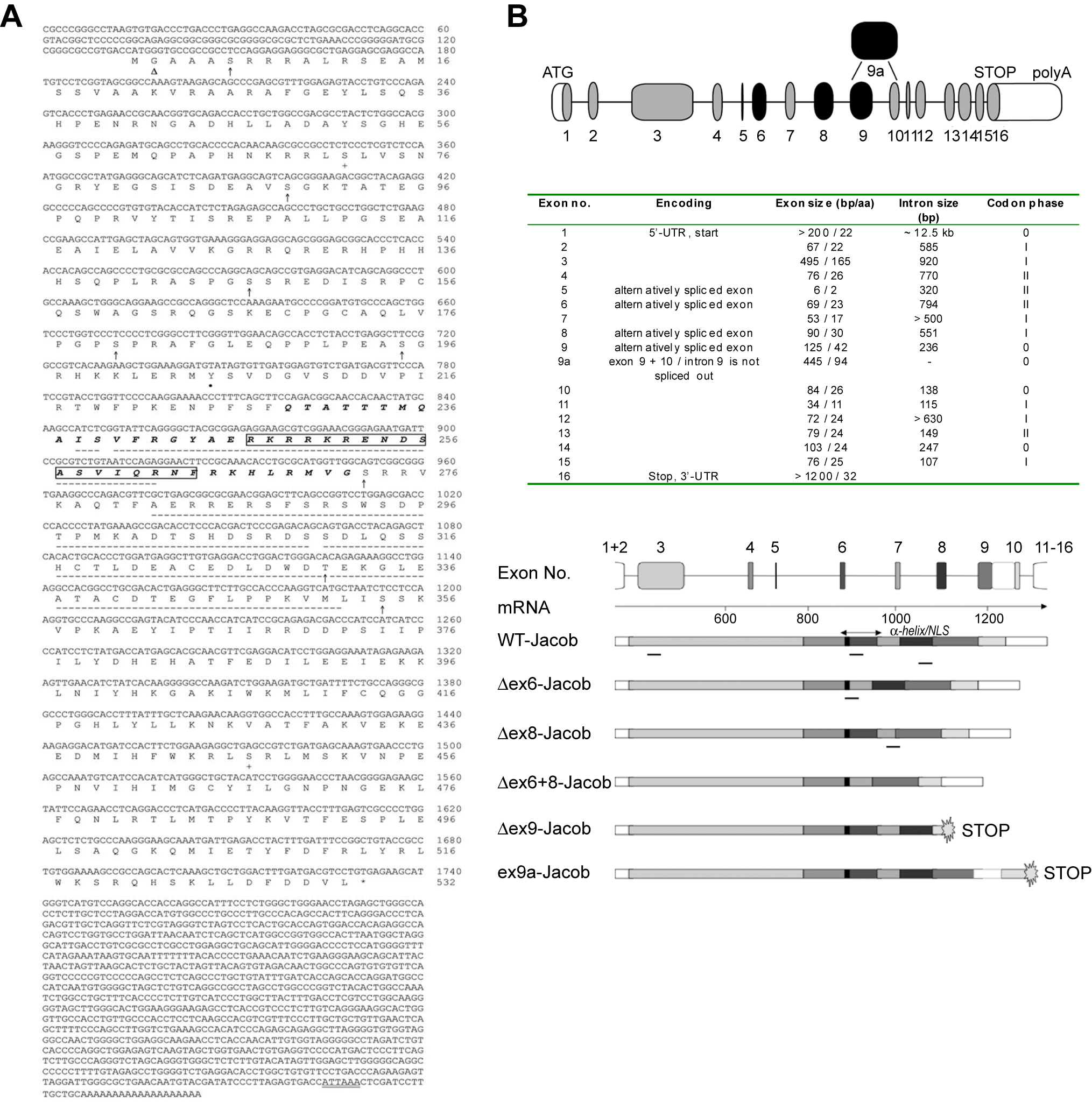

Supplement: Figure S1 — (1.5 MB TIF) [file pbio.0060034.sg001.tif]

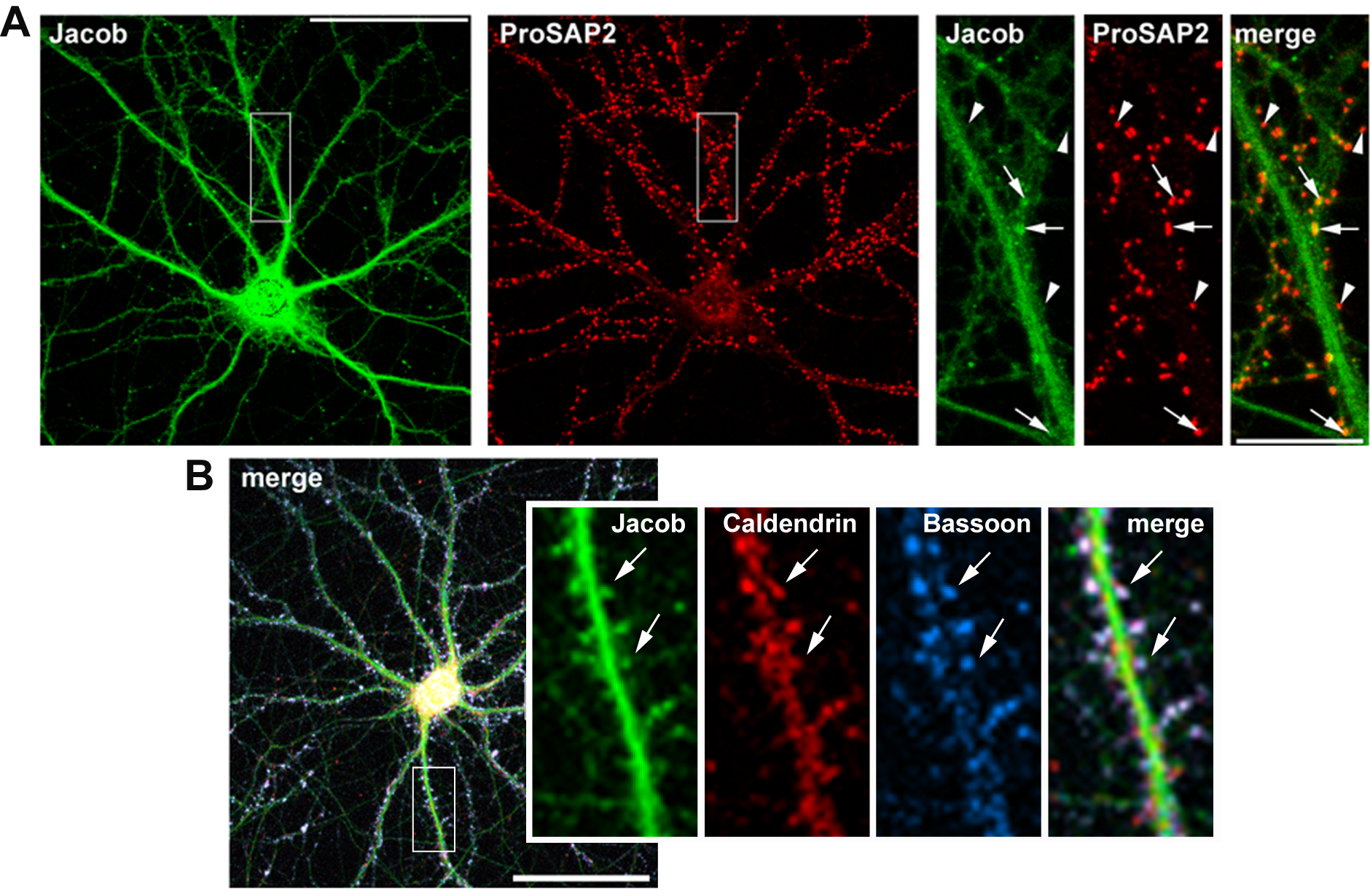

Supplement: Figure S2 — (2.2 MB TIF) [file pbio.0060034.sg002.tif]

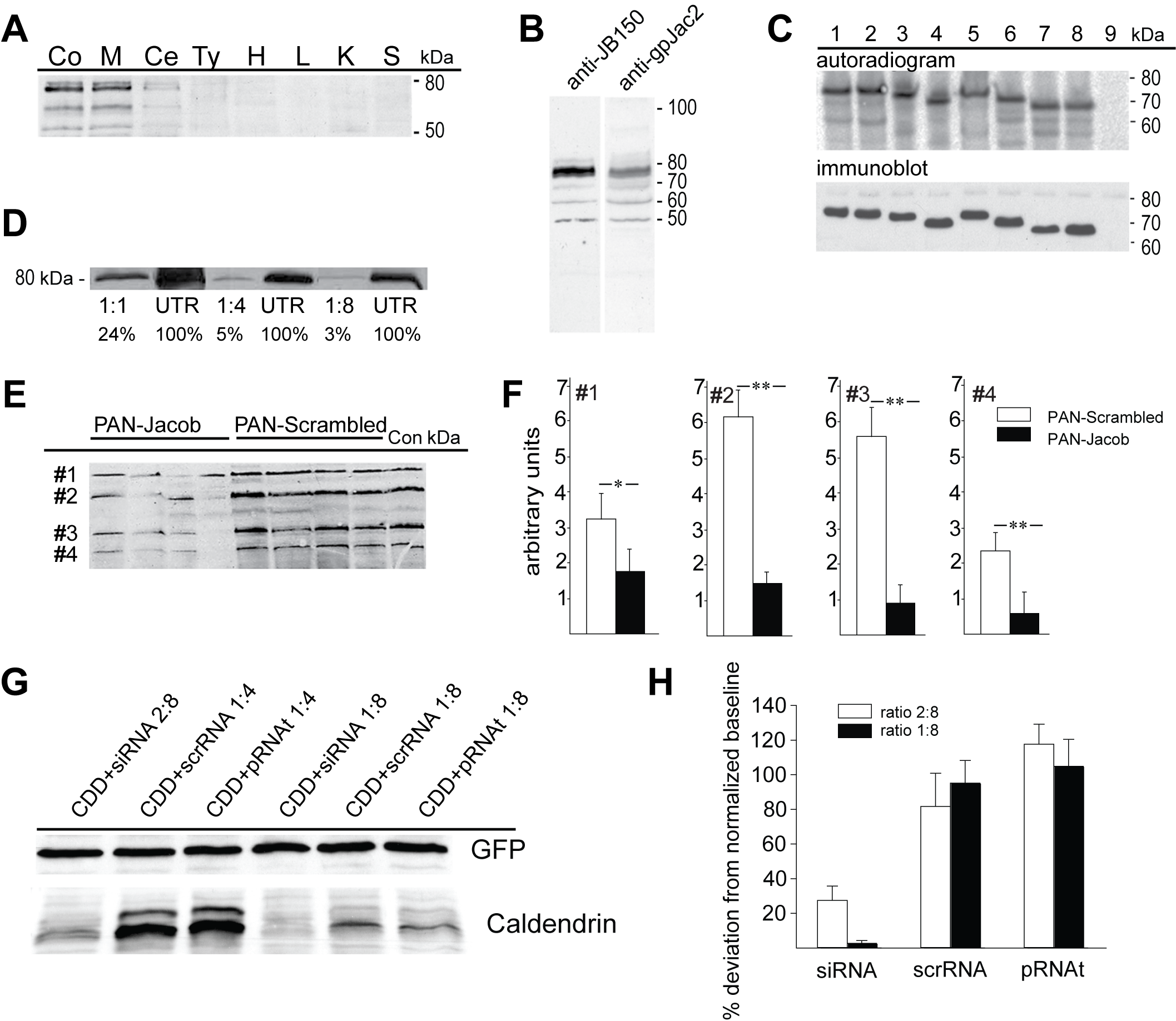

Supplement: Figure S3 — (898 KB TIF) [file pbio.0060034.sg003.tif]

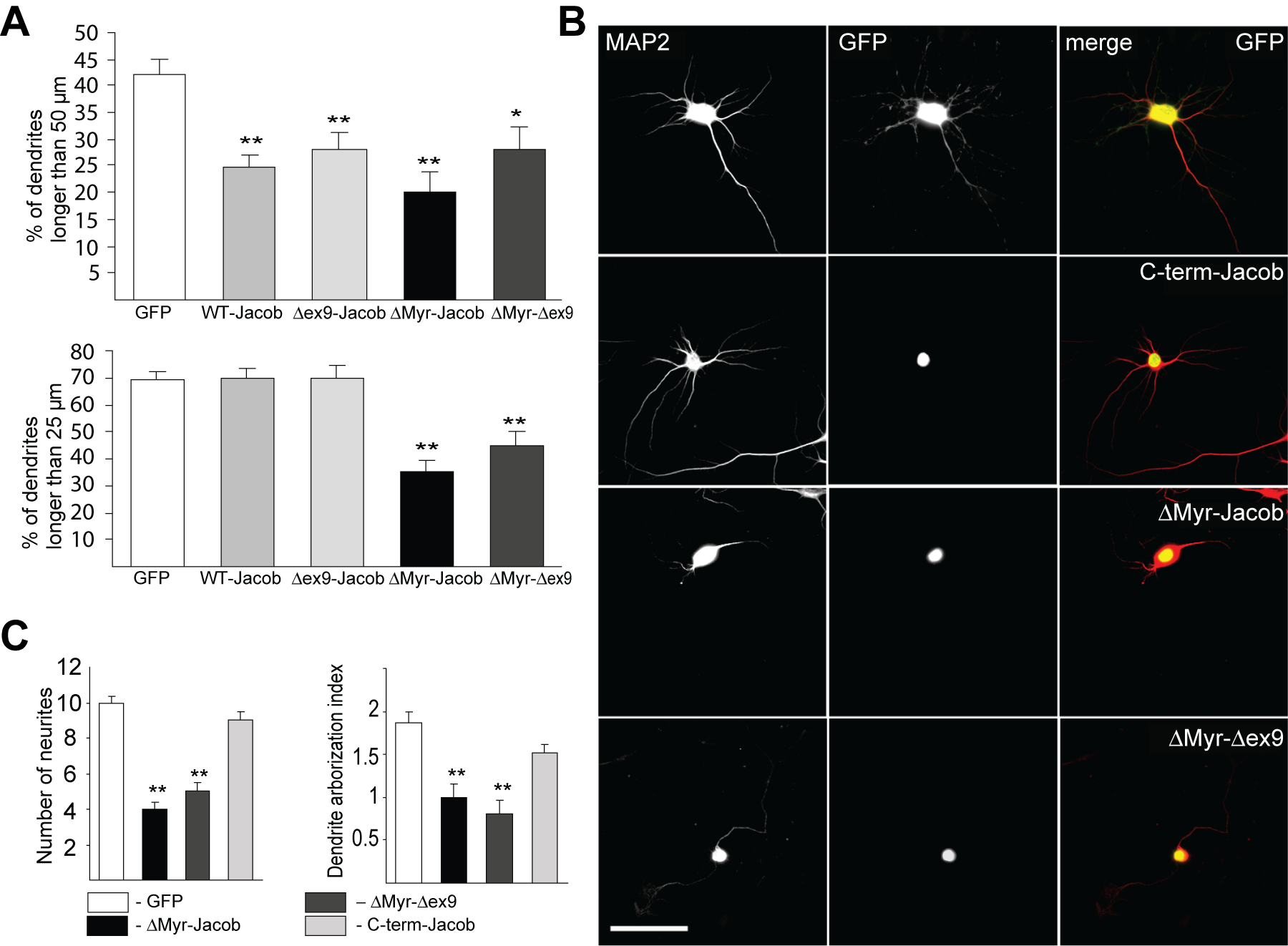

Supplement: Figure S4 — (360 KB TIF) [file pbio.0060034.sg004.tif]

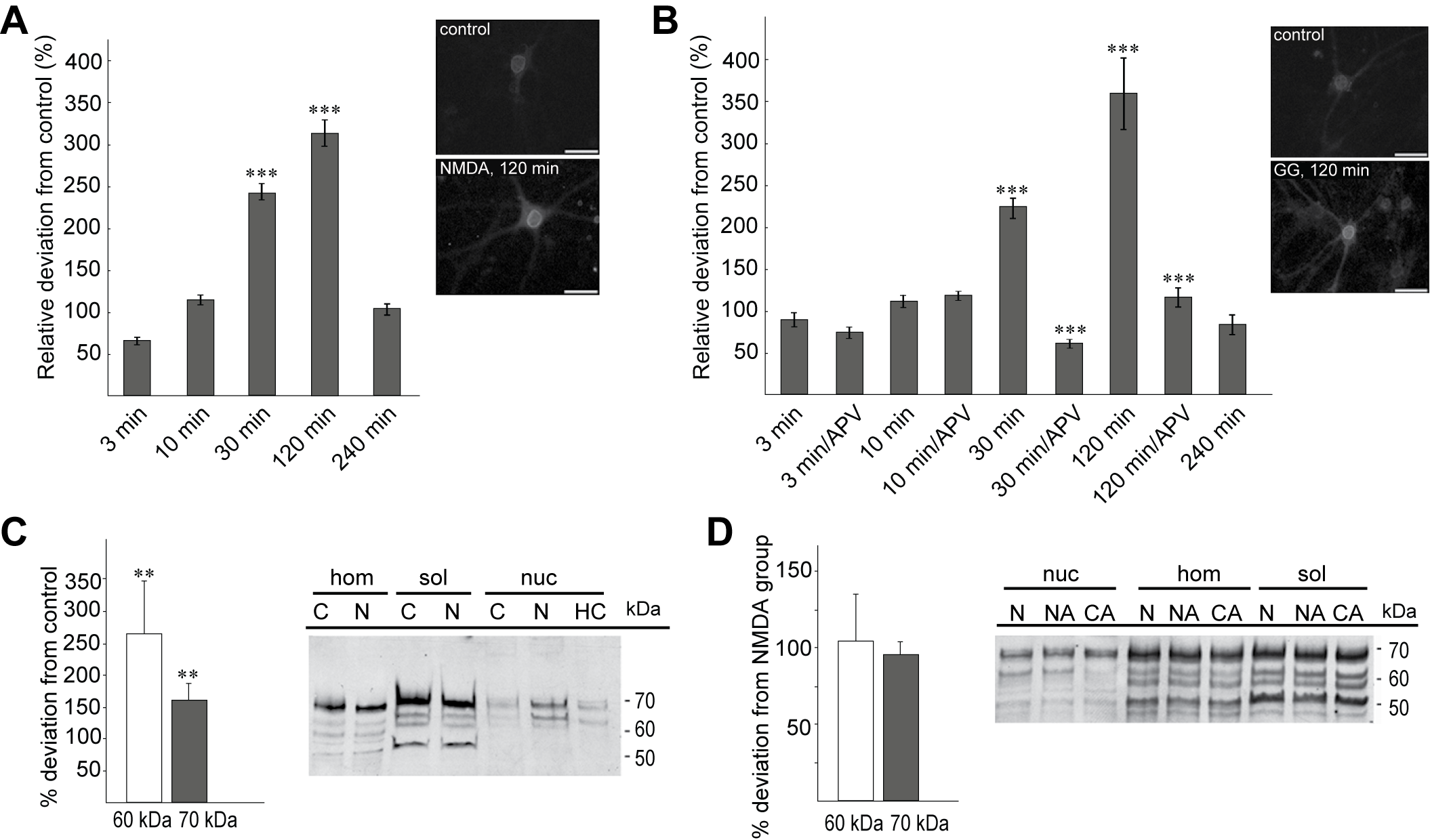

Supplement: Figure S5 — (570 KB TIF) [file pbio.0060034.sg005.tif]
