# Supplementary material for: Caldendrin–Jacob: A Protein Liaison That Couples NMDA Receptor Signalling to the Nucleus
Source: PLoS Biol. 2008 Feb 26;6(2):e34. doi: 10.1371/journal.pbio.0060034 (PMC2253627; doi:10.1371/journal.pbio.0060034)
Supplement: Text S1 — (73 KB DOC) [file pbio.0060034.sd001.doc]

**Supporting information**

**Supplementary Results**

## **Jacob Occurs as Several Isoforms with Differential Migratory Behavior in SDS-PAGE**

A polyadenylation signal was identified at nucleotide 2814 indicating that the entire 3’untranslated region is represented by the cloned Jacob cDNA (accession number AJ293697). Using the rat cDNA as a template the human Jacob gene could be identified in a DNA sequence assigned to region 9q34 of chromosome 9 (clones RP11-48C7; NT_023929.11). The gene displays a complex organization with 16 exons, most of which are smaller than 120 bp and separated by relatively small introns (Supplemental Figure 1B). Sequence alignment of human, rat and murine Jacob revealed a high degree of conservation between these species (98% identity at the amino acid level between rat and mouse; 95% rat or mouse versus human).

Interestingly, splice variants were identified that lack exon 6 resulting in Jacob isoforms that do not contain the NLS and the first amino acids of the IQ-like motif (termed ΔNLS/IQ; accession number AJ293698 / Supplemental Figure 1, 5B). Other splice variants lack the 2 amino acids encoded by the mini exon 5 (ΔIS, accession number BC006642) or display deletion of exon 8 (Δex8; accession number AJ293699) or exon 9 (Δex9; accession number AJ534642). The deletion of exon 9 results in a frameshift of the coding sequence with a stop codon at position 1145-1147 and an open reading frame coding for a 337-amino acid polypeptide. Finally, a splice isoform was identified containing the 236bp of intron 9 (termed ex9a; accession number AY255132), which also results in a premature stop codon and an open reading frame of 399-amino acids. Thus, alternative splicing can generate a multitude of different Jacob isoforms with different calculated molecular weights.

Probing of immunoblots with affinity-purified antibodies from two different species directed against a peptide sequence as well as two recombinant proteins resulted in the detection of multiple but essentially identical bands (data not shown). Jacob-immunoreactive bands could be blocked by pre-absorption of the antibodies with Jacob fusion protein (data not shown). Moreover, immunoprecipitation with antibodies generated in different species or directed against different regions of Jacob led to protein bands that were detected by antibodies not used in the immunoprecipitation experiment (data not shown). Moreover, *in vitro translation* of different Jacob splice isoform cDNAs in a cell free system results in a protein product migrating clearly above the calculated molecular weights, i.e. at about 74 kDa (Supplemental Figure 5A, B), suggesting that the conformation of the protein might also affect migration behavior in SDS-PAGE.

# Supplemental Figure legends

Supplemental Figure 1.

(A) Nucleotide and deduced amino acid sequence of Jacob; Δ marks the N-myristoylation site; potential phosphorylation sites are indicated by arrows for Protein kinase C, + for cAMP- and cGMP-dependent protein kinase and dots for Tyrosine kinase; the four alternatively spliced exons 5, 6, 8 and 9 are underlined with dashes; the central α-helical domain is indicated in bold (α-helix1: amino acids 229-247, α-helix2: 248-272), the boxed amino acids 247-264 indicate the bipartite nuclear localization signal (NLS) and the putative polyadenylation signal is marked with double lines.

(B) Genomic organization of the human Jacob gene; the four dark ellipsoids represent alternatively spliced exons, while white ellipsoids represent untranslated regions. Kramer and Wray [1] reported a murine protein termed NELF that overlaps with the N-terminal part of Jacob. The actual existence of NELF, which has been described as a secreted protein, is questionable since a stop codon that had been assigned to position 1547-1579 (Jacob cDNA), respectively bp 1425-1427 (NELF cDNA) in the original publication was corrected after submission of the Jacob sequence to public databases. The table shows all splice isoforms of Jacob, which could be identified either in cDNA library screens, in public database searches or with RT-PCR. For each splice isoform information on coding exons, apparent deletions according to the full-length cDNA (AJ293697) and the corresponding accession number is given. The scheme below illustrates the splicing events focusing on exons 3-10. The star in the scheme indicate the STOP codon in exon 10, which is due to a deletion of either exon 9 (splice isoform ∆ex9-Jacob) or insertion of intron 9 (ex9a-Jacob). All splice isoforms contain exon 1 and the N-myristoylation site.

Supplemental Figure 2.

Jacob Associates with Dendritic Spines and Co-localizes with Caldendrin

(A) Localization of Jacob (green) and the postsynaptic marker ProSAP2 (red) in 21 day-old cultured hippocampal primary neurons. Close-ups from distal dendritic arborizations reveal some colocalization of Jacob (green) and ProSAP2 (red) immunosignals in puncta decorating dendrites (arrows, yellow in the merged picture). Arrows point to postsynaptic spines lacking Jacob IR.

(B) Jacob (green) and Caldendrin (red) colocalize in postsynaptic compartments opposed to the immunolabel of the presynaptic marker protein Bassoon (blue), indicated by arrows, as well as in intradendritic patches (arrow heads) devoid of Bassoon IR. Scale bars: 50 µm (overview), 20 µm (close-ups).

Supplemental Figure 3.

(A) Quantification of neurite length for neurons transfected with the Jacob-GFP constructs depicted in Figure 4A using Scholl analysis (N: 70 cells in each group). Data are presented as mean + SEM. * p< .05; **, p< .01.

(B) Micrographs depicting MAP2/GFP labeled neurons 24 h after transfection with different Jacob-constructs. Please, note that there is no difference between the C-term-Jacob-GFP and GFP transfected cells despite the fact that C-term-Jacob-GFP is exclusively localized to the nucleus. Scale bar: 30 µm.

(C) Quantification of dendrite number and complexity of neurons transfected with the Jacob-GFP constructs depicted in B (N: 70 cells in each group). Data are presented as mean + SEM. **, p< 0.01.

Supplemental Figure 4.

(A and B) Increasing nuclear IR of endogenous Jacob in hippocampal primary neuronal cell cultures after (A) NMDA or (B) glutamate/glycine stimulation (GG) at DIV 16. NMDA and GG were applied for 3 min at 37°C. After wash-out cultures were incubated for time periods indicated and neuronal nuclei were counterstained with propidium iodide. Left panels in A and B show statistical analysis of Jacob nuclear IR at different time points after stimulation as percent deviation from fluorescence intensity of controls. Right panels show typical examples of stained control (con) and stimulated neurons. Note that exposure times are much shorter than in the previous micrographs to ease quantification. Scale bars: 50 μm.

(C and D) NMDA-stimulation of hippocampal organotypic cultures results in nuclear translocation of Jacob isoforms.

(C) Nuclear translocation of Jacob 60K and 70K in response to NMDA application. Left panel: Statistical analysis of Jacob IR in nuclear-enriched fractions after application of NMDA as percent deviation from control after densitrometric analysis of Western blots. **, p< .001. Right panel: Western blot analysis of homogenates (Hom), nuclei-free supernatants (Sol) and nuclear-enriched fractions (Nuc). C: control; N: NMDA; HC: hippocampus control. The position of size markers is indicated in kDa.

(D) Nuclear translocation occurs in the absence of protein synthesis. Left panel: Statistical analysis on IR of Jacob of nuclear-enriched fractions after application of NMDA in the presence of anisomycine as percent deviation from NMDA treatment on Western blot level (n=3).  Right panel: Western blot analysis. NA and CA indicate the presence of anisomycine. Sizes of marker proteins in kDa are indicated at the right margin.

Supplemental Figure 5.

Jacob Exists as Multiple Isoforms.

(A) Immunoprecipitation with different antibodies (anti JB150 rabbit / anti gpJac2 guinea pig) reveal that antibodies directed against different peptide sequences from different species detect the same bands on immunoblots.

(B) *In vitro* transcription/translation of the deduced Jacob ORF using a cell-free system. Western blots were loaded with *in vitro* products obtained from different Jacob splice isoforms in the vector pCMV-2c (Stratagene) for Jacob (lanes 1-8) or without: Flag-Jacob, lane 1; Flag-Δex5-Jacob, lane 2; Flag-Δex6-Jacob, lane 3; Flag-Δex8-Jacob, lane 4; Flag-Δex5+6-Jacob, lane 5; Flag-Δex5+8-Jacob, lane 6; Flag-Δex6+8-Jacob, lane 7; Flag-Δex5+6+8-Jacob, lane 8, no insert in pCMV-2c, lane 9. Upper panel: autoradiography, lower panel: immunodetection with Flag-antibody.

(C) Specificity of the antibodies was further checked by RNAi knock-down. Initially the knock-down efficiency was tested by co-transfecting COS-7 cells with the Pan-Jacob plasmid knock-down sequence and a WT-Jacob-myc-His construct followed by Western blot analysis and quantification with the Quantity One software from BioRad. Increasing the amount of vector cDNA applied during transfection as indicated in the table (Jacob: Pan-Jacob knock-down sequence) lead to a stronger knock-down of overexpressed WT-Jacob as compared to cells co-transfected with the Jacob construct and a pRNAT control. UTR, untransfected reference.

(D) Immunoblots demonstrating the knock-down of all Jacob isoforms after lentiviral infection at DIV0 of cortical primary neurons with the PAN-Jacob RNAi construct. Immunoreactivity of the four major bands (#1 - #4) is clearly reduced in protein homogenates at DIV21 after infection of cultures. A scrambled version of the targeting sequence has no effect.

(E) Quantification of the Jacob knock-down using the Quantity One software from BioRad. (N: 4) Arbritary units are presented as mean + SEM. * p< .05; **, p< .01.

(F) Knock-down of Caldendrin in Cos7 cells. Equal amounts of protein were loaded and processed with GFP or Caldendrin antibodies. Depicted are also the ratios of the amount of plasmid employed in each experiment.

(G) Quantification of the Caldendrin knock-down using the Quantity One software from BioRad. (N: 5). Data are presented as percentage deviation from normalized control levels (mean + SEM). ***, p< 0.001.

# Supplementary Material and Methods

###### Expression constructs

## WT-Jacob (aa 1-532), ΔMyr-Jacob (aa 1-532, mutation G2A), ΔNLS-Jacob (aa 1-247 + 252-532), ΔNLS/ΔMyr-Jacob (mutation G2A, aa 1-247 + 252-532), Δεx9-Jacob (aa 1-339), C-term-Jacob (aa 238-532) were subcloned into pEGFP-N1 (Clontech, Heidelberg, Germany). For *in vitro* transcription/translation, the following constructs were generated using plasmid pCMV-2c (Stratagene, Heidelberg, Germany): Flag-Jacob (bp 135-1733), Flag-Δex5-Jacob (bp 135-1733, missing bp 846-851), Flag-Δex6-Jacob (bp 135-1733, missing bp 852-919), Flag-Δex8-Jacob (bp 135-1733, missing bp 974-1062), Flag-Δex5+6-Jacob (bp 135-1733, missing bp 846-851, 852-919), Flag-Δex5+8-Jacob (bp 135-1733, missing bp 846-851, 974-1062), Flag-Δex6+8-Jacob (bp 135-1733, missing bp 846-851), 974-1062, Flag-Δex5+6+8-Jacob (bp 135-1733, missing bp 846-851, 852-919, 974-1062).

## Constructs used in the yeast two-hybrid system were as follows (all plasmids Matchmaker 2 system, Clontech, Heidelberg, Germany): WT-Jacob (aa 1-532 in pACT2 and pGBKT7), Jacob_1-230_ (aa 1-230 in pGADT7), Jacob_1-259_ (aa 1-259 in pACT2), Jacob_1-289_ (aa 1-289 in pACT2), Jacob_250-532_ (aa 250-532 in pGADT7), Jacob_212-289_ (aa 212-289 in pACT2), ΔIS-Jacob (deletion of aa 238 and 239 of the fulllength sequence in pGBKT7), ΔIS_1-260_-Jacob (deletion of aa 238 and 239, aa 1-260 in pACT2), Jacob-IQ(260,261)GG (mutation IQ260,261GG, aa 1-532 in pGBKT7), Jacob-IQ260,261GG_250-532_ (mutation IQ260,261GG, aa 253-532 in pGADT7), JacobF241E (mutation F241E, aa 1-532 in pACT2). The following GST (AP Biotech, Freiburg, Germany, plasmid pGEX-5X) and MBP (New England Biolabs, Frankfurt a.M., Germany, pMAL-2cX) constructs for Jacob were transformed into BL21 codon+ *E.coli* cells and fusion proteins were expressed and purified according to the manufacturer’s protocols: GST-J_1-230_ (aa 1-230), MBP-J_1-230_ (aa 1-230), GST-J_253-404_ (aa 253-404), GST-J_262-532_ (aa 262-532), MBP-J_262-532_ (262-532).

## Caldendrin constructs: WT-Caldendrin-GFP (aa 1-298 in pEGFP-C2), WT-Caldendrin (aa 1-298 in pACT2 and pGBKT7), Caldendrin_1-229_ (aa 1-229 in pACT2), Caldendrin_65-298_ (aa 1-229 in pACT2), Caldendrin_65-229_ (aa 1-229 in pACT2), Caldendrin_1-135_ (aa 1-229 in pACT2), Caldendrin_1-64_ (aa 1-229 in pACT2), Caldendrin_230-298_ (aa 1-229 in pACT2), Caldendrin_65-135_ (aa 1-229 in pACT2). For pull-down assays the GST-fusionprotein GST-Cald_137-298_ in pGEX-5X (AP Biotech, Freiburg, Germany) was expressed in BL21 codon+ *E.coli* cells and purified according to the manufacturer’s protocol.

## ***In vitro* Transcription/Translation**

A coupled cell-free in vitro transcription/translation system (TNT T3, Promega, Mannheim, Germany) was used to generate different Flag epitope tagged splice-isoforms of Jacob. Translation products were analyzed by SDS-PAGE, autoradiography and western blotting using a primary Flag antibody (clone M2, Stratagene, Heidelberg, Germany) and an HRP-coupled secondary antibody.

# Expression of Jacob cDNA Constructs in COS-7 Cells

COS-7 cells were grown on coverslips and transfected at 75% confluence with WT-Jacob-GFP, ΔMyr-Jacob-GFP, ΔNLS-Jacob-GFP and ΔNLS/ΔMyr-Jacob-GFP with PolyFect (Qiagen, Hilden, Germany). For analysis cells were fixated after 24 h with 4% PFA in PBS. After washing and permeabilization with 0.3% Triton X-100 for 10 min at RT, nuclei were counterstained with propidium iodide containing mounting media (Vectashield, Vector, Burlingame, USA).

**Western Blot Analysis and Subcellular Fractionation**

Tissue from adult male wistar rats was homogenized in 20 mM Tris pH 7.4 including protease inhibitors (complete® Roche, Mannheim, Germany), solubilized in SDS sample buffer and the protein concentration was determined. 20 μg of each sample were subjected to SDS-PAGE and Western blotting using Jacob primary antibodies. Subcellular fractions were prepared as described in Smalla et al. [2]. Isolation of cell nuclei from rat brain was done according to Emig et al. [3] with slight modifications. Briefly, cortices and hippocampi were homogenized in NP 0.32 (20 mM HEPES pH 7.4, containing 0.32 M sucrose, 2 mM MgCl_2_, 43 mM β-mercaptoethanol (3 μl/ml), protease inhibitor mixture (complete® Roche). Crude nuclei were pelleted by centrifugation at 180 x g for 10 min and after resuspension in NP 0.32 centrifuged again at 380 x g for 10 min. The resulting pellet was adjusted to a final sucrose concentration of 1.9 M and centrifuged at 70.000 x g for 1 h. To further increase the purity of the nuclear fraction, the pellet was resuspended in a final sucrose concentration of 2.1 M and loaded on the top of a 2.15 M sucrose layer. Nuclei were pelleted by centrifugation at 70.000 x g for 1 h and resuspended in buffer NP 0.32 without β-mercaptoethanol.

Extraction experiments of nuclei with Triton X-100, salt and chaotropic agents were performed according to Dreger et al. [4]. 500 μg crude nuclei were resuspended in 20 mM Tris-HCl, pH 7.4 containing protease inhibitor mixture (complete® Roche, Mannheim, Germany) and after addition of Benzone Nuclease (Sigma) incubated for 1.5 h at 4°C to obtain nuclear envelopes (NEs). NEs were sedimented by centrifugation (10.000 x g) for 30 min and resuspended in STM 0.25 buffer (20 mM Tris-HCl, pH 7.4, 0.25 M Sucrose, 5 mM MgCl_2_, protease inhibitor mixture (complete®, Roche). For further fractionation samples were supplemented either with Triton X-100 (final concentration 0.5% (w/v)), NaCl (final concentration 1 M), Triton X-100 and NaCl (final concentrations 0.5% (w/v) and 1 M, respectively) or with urea and Na_2_CO_3_ (final concentrations 4 M and 0.1 M, respectively). Samples were incubated for 30 min on a shaker at 4°C. Triton X-100-resistant material, as well as the NaCl-washed samples, were pelleted at 14 000 x g for 30 min. Chaotropic salt-resistant proteins were pelleted for 30 min at 100.000 x g. Pellets were re-suspended in STM 0.25 buffer. Blots were processed with the JB150 serum.

# Chromatin-Fractionation and Chromatin-Immunoprecipitation

Euchromatin fractions were prepared essentially as previously described [5]. Cell nuclei from one rat brain (approx. 1 .3 g tissue) were homogenized in 5 ml HNB (0.5 M sucrose, 15 mM Tris/HCl pH 7.5, 60 mM KCL, 0.25 mM EDTA (pH 8), 0.125 mM EGTA (pH 8), 0.5 mM Spermidine, 0.15 mM Spermin, EDTA-free Complete Protease-Inhibitor-Cocktail (Roche) with 12 strokes at 900 rpm in a Potter S homogenizer. To the homogenate 2.5 ml of HNB containing 1% Nonidet P-40 were added dropwise. After 5 min incubation at 4 °C nuclei were spun for 4 min at 1000 x g to give the supernatant S_o_ and a nuclear pellet. Nuclei were resuspended in 4.5 ml NB (20 mM Tris/HCl pH 7.5, 70 mM NaCl, 20 mM KCl, 5 mM MgCl_2_, 3 mM CaCl_2_) and digested with 50 units micrococcal nuclease (Sigma Aldrich) dissolved in 500 µl NB. Digestion was terminated by adding EDTA and EGTA to a final concentration of 5 mM each. Afterwards samples were spun at 5000 x g for 3 min to yield supernatant S1 and a nuclear pellet which was incubated for 15 min in 10 ml 2 mM EDTA. This suspension was centrifuged for 3 min at 1000 x g resulting in supernatant S2 and the Pellet P which contains the active chromatin fraction. Immunoblots were processed with the JB150 serum. For Chromatin-immunoprecipitation this pellet was rehomogenized in 5 ml Tris-buffered saline/0.1 % Triton X-100, incubated at 4 °C for 1 h and finally centrifuged for 20 min at 12000 x g. DNA was extracted with Phenol/Chloroform and after 20 PCR-cycles using hexanucleotide primers samples were loaded on agarose gels.

# Pull-down Assays

GST-Cald_137-298_ and GST for control experiments were expressed in *E. coli* strain BL21C+ (Stratagene, Heildelberg, Germany) and purified on glutathione-sepharose-4B (AP Biotech, Freiburg, Germany). A crude membrane P2 fraction from rat brain was extracted with 0.1% Triton X-100 in TBS for 30 min at 4°C and centrifuged at 20 000 x g for 30 min. The supernatant was 1:1 diluted with Ca^2+^/EGTA-buffer (1 mM) with different free Ca^2+^-concentrations (0.1 μM, 0.5 μM, 1 μM, 2 μM, 5 μM Ca^2+^) and incubated with 5 μg of either glutathione sepharose-bound GST-Cald_137-298_ or GST overnight at 4°C. After extensive washing with Ca^2+^/EGTA-buffer, bound proteins were eluted by boiling in SDS sample buffer, separated on SDS-PAGE, and analyzed by Western blotting with the JB150 antibody. For CaM-competition analysis 40 μM recombinant CaM (Calbiochem, Bad Soden, Germany) were added during overnight incubation.

# Immuno-affinity Chromatography

Total IgG from polyclonal Caldendrin (rabbit) and Jacob antiserum (rabbit, JB150) respectively were purified on GammaBind-Sepharose using FPLC according to the manufacturers instructions (AP Biotech, Freiburg, Germany) and 10 mg of these IgG fractions were transferred into coupling buffer using an FPLC Fast Desalting Column for further immobilization to 2 ml Actigel ALD (Sterogen Bioseparations, Carlsbad, USA) to yield 2 ml anti-Caldendrin coated beads and anti-Jacob coated beads respectively. Immunoaffinity chromatography was performed on FPLC HR10/2 columns. For affinity purification 15 mg of protein from a 100.000 x g supernatant of a rat brain P2 extract was loaded in buffer A (50 mM Tris/HCl, 0.5% Triton X-100, 0.2% Deoxycholate). After extensive washing with 50 total column volumes (buffer A, 100 ml) binding proteins were eluted at pH 2.5 (0.2 M Glycine/HCl, 0.5% Triton X-100), precipitated with 10% TCA, subsequently washed with acetone, lyophilized and finally solubilized in 100 µl SDS for Western blot analysis. Except for final solubilization all steps were performed at 4°C.

**Preparation of Nuclei-enriched Fractions from Hippocampal Organotypic Cultures**

Hippocampal slices from postnatal day 20 rats were prepared and cultured as described by Xiang et al. [6]. After 10 days in culture slices were first transferred to pre-warmed (34 °C) aCSF and then stimulated with 100 μM NMDA in aCSF for 15 min at 34°C. After washing twice with aCSF slices were incubated with aCSF and 100μM DL-APV for 1h at 34°C. For isolation of nuclear fractions, slices were homogenized in buffer NP 0.32. Crude nuclei were pelleted by centrifugation at 180 x g for 10 min. Pellets were resuspended in NP 0.32, centrifuged at 380 x g for 10 min, and thereafter adjusted to a sucrose concentration of 1.9 M. Purified nuclei were obtained after centrifugation at 70 000 x g for 1 h.

# Immunostainings

Sections processed for light and electron microscopy were treated identical, except that the detergent (Triton X-100) was not used for electron microscopical material. Free-floating sections were treated with 1% sodium-borohydride for 15 min and washed in PBS before incubation in a solution containing 10% normal goat serum (NGS), 0.3% Triton X-100 (optional), and 0.05% phenyl-hydrazine for 30 min. Primary antibody was diluted in 10% NGS, 0.3% Triton X-100 (optional) supplemented with 0.1% sodium azide and 0.01% thimerosal and incubated for 36h at 6°C. After washing (1h in PBS, 1h in PBS containing 0.2% bovine serum albumine = PBS-A), sections were treated with a biotinylated secondary goat-anti-rabbit antibody (Vector, Burlingame, USA, 1:2000 in PBS-A) for 24 h at 6°C. The sections were washed again and incubated for 6h with an ABC-complex (Vector Elite ABC kit, Vector, Burlingame, USA) in PBS-A. Bound peroxidase was visualized in a solution containing 1.4 mM DAB, 10 mM imidazole, 3.3 mM nickel-ammonium-sulfate, and 0.013% H_2_O_2_ in 50 mM Tris/HCl buffer. Following immunolabeling, sections for electron microscopy were transferred to 0.1 M PB and fixated with 1% osmium tetroxide in 0.1 M PB for 30 min at RT. Finally, they were dehydrated in a graded series of ethanol including a block staining with 2% uranyl acetate (Serva, Heidelberg, Germany) in 70% ethanol (10 min) and flat embedded in Durcupan. Thin silver sections were stained with uranyl acetate and lead citrate and examined using a Zeiss CEM 900.

Hippocampal primary cultures (DIV21) were fixed with Methanol at –20°C for 20 min and rehydrated in PBS before being blocked for 1 h at RT in blocking solution. Primary antibody (anti-JB150, rabbit, 1:1000; anti-ProSAP2, guinea pig, 1:500; anti-Caldendrin, guinea pig, 1:100; anti-Bassoon mAB7f, mouse, 1:2000) incubation was done overnight at 4°C in blocking solution. After PBS washing, cells were incubated with fluorescent Alexa/Cy dyes tagged secondary antibodies (1:1000, Molecular Probes / Invitrogen, Karlsruhe, Germany) and mounted in Mowiol.

**Primary Cell Culture, Transfection Studies, and Quantitative Immunocytochemistry**

Hippocampal primary cultures were prepared according to Goslin and Banker [7] using 19d rat embryos. For stimulation of primary cultured hippocampal neurons (DIV16), cells were incubated for 10 min in stimulation buffer (20 mM Na-HEPES pH 7.4, 15 mM MgCl_2_, 1.5 mM CaCl_2_, 150 mM NaCl, 5 mM KCl, 30 mM Glucose) at 37°C before treatment. 100 μM NMDA or 100 μM glutamate/10 μM glycine were applied for 3 min in stimulation buffer either in the absence or presence of the NMDA receptor antagonist DL-APV (100 μM) at 37°C. After stimulation cells were washed twice with stimulation buffer and then incubated for the indicated time points at 37°C. Cells were fixed in 100% methanol for 20 min at -20°C. After washing with PBS, cells were re-hydrated in PBS for 30 min and then incubated in blocking solution (10% horse serum, 5% sucrose, 2% BSA and 0,3% Triton X-100 in 10 mM PBS) for 30 min at RT. The primary antibody (Jacob B150) was diluted 1:100 in blocking solution and incubated overnight at 4°C. After washing (3x 10 min PBS with 0,3% Triton X-100) cells were incubated with Alexa-488 secondary goat-anti-rabbit antibody (1:1000 in blocking solution) for 2 h at RT, washed again (2x 10 min PBS with 0,3% Triton X-100; 1x 10 min PBS) and embedded in Vectashield (Vector, Burlingame, USA), containing propidium iodide for nuclear staining. Images were taken using a Leica DMRXE microscope and a Spot RT camera (Visitron Systems). Images were analyzed using NIH image, version 1.57. The following other antisera were used: rabbit anti-synapsin (Synaptic Systems), monoclonal anti-MAP2 (Sigma), rabbit anti-phospho-CREB (Ser 133) (Upstate), mouse anti-Importin-α1/Karyopherin-α/Rch-1 (BD Transduction Laboratories), mouse anti-GFP (Babco), rabbit anti-Caldendrin (Seidenbecher et al., 1998).

**Morphological Analysis of Transfected Hippocampal Neurons**

For transfection, 105 μl of Transfection Mix Solution 1 (7 µg DNA, 253.3 mM CaCl_2_) was mixed with the same volume of 2x HBS (42 mM HEPES pH 7.01, 247 mM NaCl, 10 mM KCl, 1.4 mM Na_2_HPO_4_, 15 mM Glucose). 180 µl of this reaction was then mixed with 7.5 µl NeuroPorter (PeqLab, Erlangen, Germany) and 30 µl Neurobasal (Invitrogen, Invitrogen, Karlsruhe, Germany) and incubated at RT for 20 min. 72.5 μl of this mix was added dropwise to the cells in OptiMEM I medium (Invitrogen, Karlsruhe, Germany). After 2 h medium was changed to Neurobasal growth medium. Cells were fixated in 100% methanol for 20 min at -20° C. After washing, cells were re-hydrated in PBS and then incubated in blocking solution for 30 min at RT. The primary antibodies anti-MAP2 (Sigma, Munich, Germany), anti-GFP (rb290, Abcam, Hiddenhausen, Germany), anti-Bassoon (mAB7f) were diluted 1:500, 1:2000, and 1:1000 respectively in blocking solution and incubated for 36h at 4°C. After washing (3x 10 min in PBS) cells were incubated with Alexa-568 (goat-anti-mouse) or Alexa-488 (goat-anti-rabbit) secondary antibody (both Molecular Probes/Invitrogen, Karlsruhe, Germany, 1:1000 in blocking solution) for 2 h at RT. After washing with PBS cells were embedded in Mowiol. Images were taken using a Zeiss-AxioplanII-imaging-fluorescent microscope, Spot RT camera and MetaView program (Visitron System) and analyzed using Adobe Photoshop (Version 8.0). 100 neurons were sampled randomly for each experimental condition.

**Cell death assays**

Primary hippocampal neurons were transfected with RNAi-NLS-GFP (DIV8) to knock-down nuclear Jacob or with a pRNAt vector control and then stimulated (DIV15) with 100 µM NMDA for 3 min or with 300 µM NMDA for 5 min. 24 hours after NMDA exposure dead cells were determined by nuclei staining with 100 ng/ml DAPI and propidium iodide PI (10 µM), respectively. After 30 min incubation with DAPI and PI neurons were fixed and ration between dead and total number of cells was determined. To determine apoptotic cells, neurons were fixed 24 hours after NMDA exposure and then stained following the protocol of the ApopTag ® Red Apoptosis Detection Kit (Chemicon). DNA stand breaks were detected by enzymatic labeling the free 3’-OH termini with digoxigenin conjugated nucleotides and visualized by using an anti-digoxigenin conjugated-rhodamine antibody. Neuronal nuclei were counterstained with DAPI.

# Confocal Laserscan Microscopy

Synaptic glutamate receptors were stimulated with bath application of bicuculline (50µM) and 4-AP 2.5 mM for 30min. Synaptic NMDA receptors were irreversibly blocked with the co-administration of 5 µM MK-801. Activation was induced by bath application of NMDA (100µM for 3 min / Sigma) after extensive washing of the cultures that were treated with MK-801 as outlined above. Cultures were then incubated for 30 min before fixation and subsequent immunostaining for pCREB, Jacob and Importin-α. To block NR2B containing NMDA receptors ifenprodil (5 µM / Sigma) was applied in parallel to NMDA.

The region of interest was scanned with a confocal laser scanning microscope (Leica M IRE2, Leica Bensheim, Germany) equipped with a Krypton-Argon-Ion laser (488/568/647 nm) and an acousto-optic-tunable filter (AOTF) for selection and intensity adaptation of laser lines. The configuration of the system (excitation beam splitter: TD (488/568), detector beam splitter: RSP 580, barrier filter: BP 535 (channel 1) and detector beam splitter: RSP 660, barrier filter: BP 600 (channel 2) and barrier filter OG 665 (channel 3) allowed a simultaneous or sequential detection of fluorescence emission from the dyes (Molecular Probes / Invitrogen, Heidelberg, Germany) Alexa 488 (indicative for Jacob Immunofluorescence), Alexa 568 (indicative for Caldendrin or ProSAP2 Immunofluorescence) and Cy5 (indicative for Bassoon-Immunofluorescence) without crosstalk. In case of simultaneous scanning the intensity of the laser lines and the voltage of the photomultipliers were adjusted to values that excluded any bleeding of the dyes across channels. To make sure that no crosstalk between dyes was detected during image acquisition all images were scanned sequentially. Along the z-axis usually 7 to 10 thin optical sections with a z-resolution of 0.6 to 1 µm (focus depth) were scanned at different magnifications in a 1024 x 1024 pixel format. Usually, images were taken with a Fluotar 25x oil NA 0.75, Fluotar 40x oil NA 1.0-0.5, a Plan Apo 63x oil NA 1.4 or Plan Apo 100x oil NA 1.4 as objetive lens at various zoom factors as indicated in the legends. Subsequently, maximum intensity projections (extended focus images) were calculated from each fluorescence channel of the image-stack and stored as RGB images together with the original image stacks. Image analysis was carried out with Adobe Photoshop (Version 7.0; Adobe Systems Inc., Mountain View, CA, USA).

Jacob and Importin nuclear IR quantitative assays were performed with Image J software (http://rsb.info.nih.gov/ij/) using primary hippocampal cultures at DIV16. The region of interest (ROI) for the nuclei were determined using the threshold from DAPI staining, subsequently saved in ROI manager and later on applied for the correspondent images with anti Jacob and anti Importin staining. The nuclear Jacob and Importin IR was measured as mean grey value (in a.u. of pixel intensity) and differences between groups as relative deviation from control were calculated.

To determine the Jacob nuclear overexpression pCREB levels primary hippocampal neurons (Density: 40000 cells per coverglass) were transfected with C-term-Jacob-GFP and ΔMyr-Jacob-GFP constructs at DIV11, fixed the next day, stained with MAP2- (Sigma) and pCREB-specific antibodies (Ser133 / Upstate) and mounted with DAPI containing medium. For the analysis of the CREB phosphorylation level we used maximal projection images calculated from z-stacks. Thus, the boarders of the each stack were defined based on MAP2 staining in order to obtain the whole depth of the nucleus. The ROIs for the nuclei were determined using the threshold of DAPI staining. The level of pCREB fluorescence was measured in transfected and non-transfected neurons as pixel intensity and calculated as relative deviation from control.

**Time Lapse Live Cell Imaging**

Temporal dynamics of the nuclear translocation of Jacob in hippocampal cultures were studied using CLSM equipped with a Krypton- Argon-Ion laser (Leica TCS 4D, Leica Bensheim, Germany). Neurons transfected with GFP tagged WT-Jacob and ΔNLS-Jacob constructs were imaged at 2 min intervals. Immediately prior to image acquisition cultures were transferred to a temperature-controlled microscope chamber filled with a pre-warmed 37 °C oxygenated bath solution (pH=7.3) containing 129 mM NaCl, 5 mM KCl, 2 mM CaCl2x2H2O, 1 mM MgCl2x6H2O, 30 mM glucose, 20 mM HEPES and 7.5µM anisomycin.

Images were acquired using the Leica TCS software. In order to minimize photobleaching of the sample scanning was performed at medium scan mode (speed) with 512 x 512 pixel resolution, two-times line averaging and with laser intensity set 15%. Fluorescence changes were quantified from averaged confocal image stacks (7 planes) acquired using ImageJ software in the regions of interest. The distance between the first and the last plane was between 5-6 µm. To examine whether Jacob translocates from distal dendrites into the nucleus we also quantified the intensity of dendritic fluorescent after glutamate stimulation of neurons. Fluorescent changes (Fluorescence intensity values) are presented as percentage change from baseline and normalized for fluctuations in whole image intensity values.

# Computer Modeling

The suggested model structure is based on coordinates from protein data bank, [8] entry 1cdm, which has the highest sequence homology to Caldendrin (41% identical, 27% similar). Model structures of the entire Caldendrin binding region were depicted using coordinates from protein structures, representing the “open” (1cdm, 1g4y, 1exr), the “closed” (1wdc), or the “semi-open” (1wdc, 1aji, 1iwq) conformation of CaMs. The structures were modeled using Swiss-PDB Viewer [9], Modeller [10], and Threader [11], and were visualized using PyMol X11 and O.

**Supplementary references**

1. Kramer PR, Wray S (2000) Novel genes expressed in nasal region influences outgrowth of olfactory axons and migration of luteinizing hormone-releasing hormone (LHRH) neurons. Genes Dev 14:1824-1834.

2. Smalla KH, Seidenbecher CI, Tischmeyer W, Schicknick H, Wyneken U et al. (2003) Kainate-induced epileptic seizures induce a recruitment of caldendrin to the postsynaptic density in rat brain. Mol Brain Res 116:159-162.

3. Emig S, Schmalz D, Shakibaei M, Buchner K (1995) The nuclear pore complex protein p62 is one of several sialic acid-containing proteins of the nuclear envelope. J Biol Chem 270:13787-13793.

4. Dreger M, Bengtsson L, Schoneberg, T, Otto H, Hucho F (2001) Nuclear envelope proteomics: novel integral membrane proteins of the inner nuclear membrane. Proc. Natl. Acad. Sci. U S A. 98:11943-11948.

5. Reyes JC, Muchardt C, Yaniv M (1997) Components of the human SWI/SNF complex are enriched in active chromatin and are associated with the nuclear matrix. J Cell Biol 137:263-274.

6. Xiang Z, Hrabetova S, Moskowitz SI, Casaccia-Bonnefil P, Young SR et al. (2000) Long-term maintenance of mature hippocampal slices *in vitro*. J Neurosci Methods 98:145-54.

7. Goslin K, Banker G (1991) Rat hippocampal neurons in low-density culture. In Culturing nerve cells (G. Banker and K. Goslin, Eds.), pp.251-282. MIT Press, Cambridge, MA.

8. Berman HM, Westbrook J, Feng Z, Gilliland G, Bhat TN et al. (2000) The Protein Data Bank. Nucleic Acids Res 28:235-242.

9. Guex N, Peitsch MC (1997) SWISS-MODEL and the Swiss-PdbViewer: an environment for comparative protein modeling. Electrophoresis 18:2714-2723.

10. Sali A, Potterton L, Yuan F, van Vlijmen H, Karplus M (1995) Evaluation of comparative protein modeling by MODELLER. Proteins 23:318-326.

11. Jones DT, Taylor WR, Thornton, J.M. (1992) A new approach to protein fold recognition. Nature 358:86-89.
